# Utilization of diagnostic resources and costs in patients with suspected cardiac chest pain

**DOI:** 10.1093/ehjqcco/qcaa064

**Published:** 2020-08-18

**Authors:** Marijke P M Vester, Daniëlle C Eindhoven, Tobias N Bonten, Holger Wagenaar, Hendrik J Holthuis, Martin J Schalij, Greetje J de Grooth, Paul R M van Dijkman

**Affiliations:** Department of Cardiology, Leiden University Medical Center, PO Box 9600, 2300 RC Leiden, The Netherlands; Department of Public Health and Primary Care, Leiden University Medical Center, PO Box 9600, 2300 RC Leiden, The Netherlands; Performation-HOTflo, Sweelincklaan 1, 3723 JA, Bilthoven, The Netherlands; Performation-HOTflo, Sweelincklaan 1, 3723 JA, Bilthoven, The Netherlands; Department of Cardiology, Leiden University Medical Center, PO Box 9600, 2300 RC Leiden, The Netherlands; Department of Cardiology, Leiden University Medical Center, PO Box 9600, 2300 RC Leiden, The Netherlands; Department of Cardiology, Leiden University Medical Center, PO Box 9600, 2300 RC Leiden, The Netherlands

**Keywords:** Chest pain, Angina pectoris, Quality improvement, Cost-effectiveness

## Abstract

**Aims:**

Non-acute chest pain is a common complaint and can be caused by various conditions. With the rising healthcare expenditures of today, it is necessary to use our healthcare resources effectively. This study aims to give insight into the diagnostic effort and costs for patients with non-acute chest pain.

**Methods and results:**

Financial data of patients without a cardiac history from four hospitals (January 2012–October 2018), who were registered with the national diagnostic code ‘no cardiac pathology’ (ICD-10 Z13.6), ‘chest wall syndrome’ (ICD-10 R07.4), or ‘stable angina pectoris’ (ICD-10 I20.9) were extracted. In total, 74 091 patients were included for analysis and divided into the following final diagnosis groups: no cardiac pathology: *N* = 19 688 (age 53 ± 18), 46% male; chest wall syndrome: *N* = 40 858 (age 56 ± 15), 45% male; and stable angina pectoris (AP): *N* = 13 545 (age 67 ± 11), 61% male. A total of approximately €142.7 million was spent during diagnostic work-up. The total expenditure during diagnostic effort was €1.97, €8.13, and €10.7 million, respectively for no cardiac pathology, chest wall syndrome, and stable AP per year. After 8 years of follow-up, ≥95% of the patients diagnosed with no cardiac pathology or chest wall syndrome had an (cardiac) ischaemic-free survival.

**Conclusion:**

The diagnostic expenditure and clinical effort to ascertain non-cardiac chest pain are high. We should define what we as society find acceptable as ‘assurance costs’ with an increasing pressure on the healthcare system and costs.

## Introduction

Chest pain may be a symptom of ischaemic heart disease.^1^ Every year 0.7–2.7% of the general population consult the general practitioner for having chest pain.^2–5^ Multiple underlying causes have been described varying from a musculoskeletal origin, gastro-oesophageal reflux disease to potential life-threatening events such as coronary artery disease (CAD). Fortunately, only a minority of patients have chest pain symptoms due to ischaemic heart disease.^6^

Because of limited diagnostic resources in a primary care setting, patients suspected of a cardiac cause of chest pain are often referred to a cardiologist for additional diagnostic testing. However, of the referred patients with chest pain, no cardiac cause has been found in 55–90%.^5^^,^^7^ As the health expenditures are increasing, so does the need to spend the available resources sparingly.^8^ Ideally, healthcare providers aim to offer the highest quality of care and use the minimal required additional diagnostic procedures to make the correct diagnosis. On the other hand, in the diagnostic trajectory of a patient with chest pain, a wide variety of diagnostic tests are available and it appears that besides the use of guidelines, the choice of diagnostics depends on the opinion of the consultant doctor and the location of the consultation.^9–11^

Numerous studies have investigated the final diagnosis of patients with chest pain in primary care.^2^^,^^3^^,^^5^^,^^12^ However, to our knowledge, studies examining the healthcare trajectory of patients with non-acute, or chronic, chest pain in a hospital setting are scarce. A recent study verified that financial databases are a valid source of information in the evaluation of a patient’s care chain.^13^ Given the high prevalence of chest pain and high healthcare expenditures on CAD, investigating this care chain is of relevance for the everyday cardiology practice. The current study aims to gain insight into the amount of referred patients, utilization of diagnostic resources and costs, and clinical outcome in patients with non-acute chest pain in a hospital setting using financial data.

## Methods

### Patient population and data sources

All Dutch citizens are covered by a basic mandatory insurance. Treatments and diagnoses supplied by health services are coded according to a national financial coding system [DOT: Diagnose Behandeling Combinatie (DBC) Op weg naar Transparantie] combined with the World Health Organization (WHO) International Classification of Diseases (ICD). Declaration data from unique patients of 18 years and older with a new onset of suspected cardiac chest pain seen in the outpatient clinic were extracted from the financial database of Performation-HOT*flo* from January 2012 until October 2018.

Performation-HOT*flo* (Bilthoven, the Netherlands) is a healthcare consultancy company that provides patient-level costing and benchmarking products for different healthcare services across Europe.^14^ Hospital selection was based on the region of South-Holland (Zuid-Holland) and the availability of cost-price information by Performation-HOT*flo*. The relevant hospitals were requested to give consent for using their data for this study. Four hospitals, of which one academic hospital and three regional hospitals, gave consent.

Suspected cardiac chest pain was defined by the following three diagnosis codes ‘no cardiac pathology’ (DOT code 0320.101 similar to ICD-10 Z13.6), ‘chest wall syndrome’ (DOT code 0320.201 similar to ICD-10 R07.4), or ‘stable angina pectoris’ (DOT code 0320.202 similar to ICD-10 I20.9). The diagnosis code was recorded after the first diagnosis. Subsequently, patients with an ischaemic cardiac history or with another cardiac history were excluded from the analysis. The remaining patients were divided into three different groups based on diagnosis code:

- Group I: no cardiac pathology. Also defined as chest pain of no cardiac origin.
- Group II: chest wall syndrome. Also defined as chest pain of no cardiac origin.
- Group III: stable angina pectoris (AP). Also defined as cardiac chest pain.

In a combination of two or more of previously mentioned diagnosis codes within the same patient, the code stable AP had priority above chest wall syndrome and no cardiac pathology. The code chest wall syndrome had priority above no cardiac pathology.

### Activities

The following characteristics were retrieved after first diagnosis registration: age, gender, and all healthcare utilization with the associated admission. To gain more insight into the care process and expenditures, the used resources were divided into the following categories: ‘Cardiac Invasive Diagnostics or Treatment’, ‘Cardiac non-invasive diagnostics’, ‘Emergency Department’, ’Inpatient care’, ‘Outpatient care’, and ‘Other’. Other included, i.e. the use of materials and administrative costs (Supplementary material online, *Table S1*).

### Cost analysis

By using time-driven activity-based costing (TD-ABD) methodology costs were calculated at patient-level resource utilization.^15^TD-ABD is a micro-costing method, and calculates two parameters per activity: the costs per time unit to perform each activity and the overall time units spent performing the activity. As cost price calculations are standardized by Performation-HOT*flo*, it was possible to compare participating hospitals. The database contained information about the period of treatment, the differently registered diagnostics and the registered interventions. All available data from January 2012 until October 2018 were obtained and the most recent cost price model was used for calculations. Therefore, differences in the cost price calculations due to inflation were avoided.

### Statistical analyses

Continuous data are presented as mean and 95% confidence interval (CI) when normally distributed. Categorical data are presented as numbers and percentages. A χ^2^ test was used for comparing the baseline characteristics of the different patient groups. *P*-values <0.05 were considered statistically significant. The duration of follow-up was calculated from the date when the first diagnosis code was registered, the inclusion of the patient until the last date activity for one of the above-mentioned diagnosis or resource code was registered. The time to an ischaemic event after the inclusion of a patient is presented in a Kaplan–Meier plot. An ischaemic event was based on diagnosis codes and resource use after the first registration:

- Unstable AP or infarction: patients who developed unstable AP (DOT code 0320.11.203 similar to ICD-10 I20.0), a non-ST-segment elevation myocardial infarction (DOT code 0320.11.205 similar to ICD-10 I21.4), an ST-segment elevation myocardial infarction (DOT code 0320.11.204 similar to ICD-10 I21.9) or who were followed up after acute coronary syndrome (DOT code 0320.11.801 similar to ICD-10 Z86.7).
- Percutaneous coronary intervention (PCI) or coronary artery bypass grafting (CABG): patient who underwent a percutaneous coronary intervention or a CABG (DOT code 0320.11.802 or 0320.11.810 similar to ICD-10 Z09.0) and the cardiothoracic declaration codes from the Performation Hot*flo* database [Cardiothoracic (CTC) codes for a CABG coded as 2320, 2400, 2415, 2425, or 2470 similar to ICD-10-PCS codes based on procedure].

Statistical analyses were performed using SPSS (version 23.0, IBM SPSS Statistics).

### Ethical considerations

Performation-Hot*flo* is ISO 27001 and NEN 7510 certified, meaning that all patient data are used in strict confidence. All members of the research team signed a statement of confidentiality for processing the data. The participating hospitals formally consented to participate. The local medical ethics committee of the Leiden University Medical Center, in its capacity as the coordinating centre of this retrospective study, approved the study design and waived the need for individual patient informed consent.

## Results

### Population

**Figure 1:**
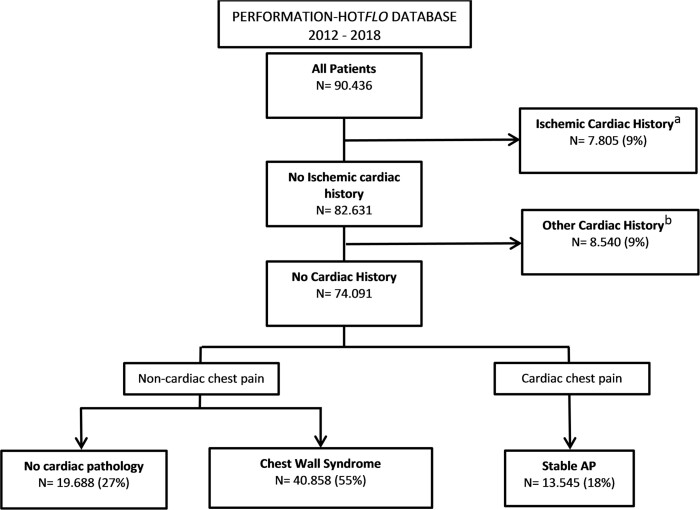
Flow chart of included patients. Patients were subdivided into groups, based on the different diagnostic codes: no cardiac pathology’ (coded as 0320.101 similar to ICD-10 Z13.6), idiopathic thoracic complaints (coded as 0320.201 similar to ICD-10 R07.4), and stable angina pectoris (AP) (coded as 0320.202 similar ICD-10 I20.9). ^a^Ischaemic cardiac history: see Supplementary material online, *Table S1*. ^b^Other cardiac history: see Supplementary material online, *Table S2*.

In total, 90 436 unique patients in four hospitals between January 2012 and October 2018 were included. Patients with a history of ischaemic cardiac disease (*N* = 7805, 9%) or another cardiac history (*N* = 8540, 9%) were excluded for analysis. Subsequently, a total of 74 091 (mean age 57 ± 16, 48% men) with no cardiac history and new onset of cardiac chest pain remained. Of these patients, 19 688 (27%) had no cardiac pathology, 40 858 (55%) had chest wall syndrome, and 13 545 (18%) had stable AP as the final diagnosis (*Figure 1*). The stable AP patients (age 67 ± 11, men 61%) were older and consisted of more men in comparison to both other patient groups [non-cardiac chest pain, no cardiac pathology (age 53 ± 18, 46% men), and chest wall syndrome (age 56 ± 15, 45% men)] (*Table 1*).

**Table 1:** Baseline characteristics

	No cardiac pathology, *N* = 19 688 (27%)	Chest wall syndrome, *N* = 40 858 (55%)	Stable AP, *N* = 13 545 (18%)	Total, *N* = 74 092	*P*-value
Age (mean ± SD)	53 ± 18	56 ± 15	67 ± 11	57 ± 16	<0.001
Gender (*n*, % male)	8971 (46%)	18 477 (45%)	8203 (61%)	35 651 (48%)	<0.001

Stable AP, stable angina pectoris

### Activities and costs

**Figure 2:**
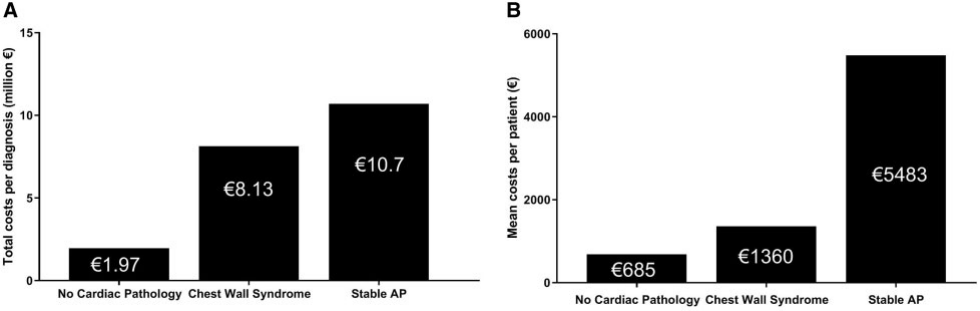
A total of €142 702 110 (2011–2018) was spent between January 2012 and October 2018. (*A*) The total costs in the group with no cardiac pathology (*N* = 19 688), chest wall syndrome (*N* = 40 858), and stable AP (13 545) in millions (€) per year. (*B*) The mean costs (€) per patient per group. Stable AP, stable angina pectoris.

A total of €142 702 110 was spent during the diagnostic work-up period. The group with no cardiac pathology covered 9% (€13 485 941) of the total costs, the group with chest wall syndrome 39% (€55 557 410). The majority of the expenditures were covered by the group with stable AP (52%, €73 658 759). *Figure 2A* shows the overall costs per year per diagnosis: €1 973 552 in the no cardiac pathology group, €8 130 352 chest wall syndrome group, and €10 779 330 in the stable AP group. This corresponds with a mean expenditure of €685, €1360, and €5483 per patient with no cardiac pathology, chest wall syndrome, and stable AP, respectively (*Figure 2B*). As is shown in *Table 2*, most money was spent on ‘Inpatient Care’(€50 428 212, 35%) followed by ‘Cardiac non-invasive diagnostics’(€39 924 124, 28%), ‘Cardiac invasive diagnostics or treatment’(€28 627 656, 20%), ‘Outpatient Care’ (€13 535 585, 10%), ‘Emergency Department’ (€8 275 256, 6%), and ‘Other’ €1 911 276, 1%). The no cardiac pathology group and the chest wall syndrome group spent more money in the emergency department, compared with the stable AP group. A possible explanation could be that because of more uncertainty and concern these patients are referred to the emergency department to rule out an acute coronary syndrome. *Table 3* shows the percentage of patients per group who underwent non-invasive diagnostic tests. In all groups, almost every patient underwent a non-invasive diagnostic test. The most applied non-invasive diagnostic test was an electrocardiogram (ECG) in no cardiac pathology, chest wall syndrome, and stable AP, 80%, 87%, 79%, respectively. Followed by laboratory tests in the group with chest wall syndrome (76%) and stable AP (76%) and cardiac ultrasound in the group with no cardiac pathology (54%). The proportion of applied diagnostics in the groups with chest wall syndrome and stable AP were comparable with each other.

**Table 2:** Overview of healthcare expenditures per group

Category (*N*, %)^a^	No cardiac pathology, *N* = 19 688 (27%)	Chest wall syndrome, *N* = 40 858 (55%)	Stable AP, *N* = 13 545 (18%)	Total
Cardiac invasive	€1 112 551 (8)	€4 054 831 (7)	€23 460 274 (32)	€28 627 656 (20)
Cardiac non-invasive	€4 945 849 (37)	€19 902 132 (36)	€15 076 143 (21)	€39 924 124 (28)
Emergency department	€1 682 815 (13)	€4 881 403 (9)	€1 711 038 (2)	€8 275 256 (6)
Inpatient care	€3 498 977 (26)	€20 832 042 (38)	€26 097 193 (35)	€50 428 212 (35)
Outpatient care	€2 142 629 (16)	€5 743 350 (10)	€5 649 606 (8)	€13 535 585 (10)
Other	€103 119 (1)	€143 653 (0)	€1 664 504 (2)	€1 911 276 (1)
Total	€13 485 941 (9)	€55 557 410 (39)	€73 658 759 (51)	€142 702 110 (100)

Stable AP, stable angina pectoris.

^a^ Distinction of the different categories can be seen in Supplementary material online, *Table S1*.

**Table 3:** The amount of patients (in percentages) that underwent a non-invasive diagnostic test per group

	No cardiac pathology, *N* = 19 688 (27%)	Chest wall syndrome, *N* = 40 858 (55%)	Stable AP, *N* = 13 545 (18%)
Non-invasive diagnostics (*N*, %)	18 783(90)	40 461 (99)	12 545 (93)
ECG	15 682 (80)	35 646 (87)	10 734 (79)
Cardiac ultrasound	10 678 (54)	16 216 (40)	8672 (64)
Laboratory	7762 (39)	30 857 (76)	10 232 (76)
Exercise test	3780 (19)	19 638 (48)	7217 (53)
X-ray	2562 (13)	14 986 (37)	5628 (42)
Holter monitor	2440 (12)	3907 (10)	1731 (13)
Cardiac CT scan	1233 (6)	9845 (24)	3256 (24)
Cardiac nuclear/SPECT scan	510 (3)	3585 (9)	2787 (21)
Rhythm monitoring	469 (2)	4154 (10)	2732 (20)
Cardiac MRI	268 (1)	1793 (4)	1331 (10)
ABPM	135 (1)	62 (2)	447 (3)

ABPM, ambulatory blood pressure monitoring; Cardiac CT scan, cardiac computed tomography scan; ECG, electrocardiogram; MRI, magnetic resonance imaging; SPECT, single-photon emission computed tomography; Stable AP, stable angina pectoris.

### Follow-up

**Figure 3:**
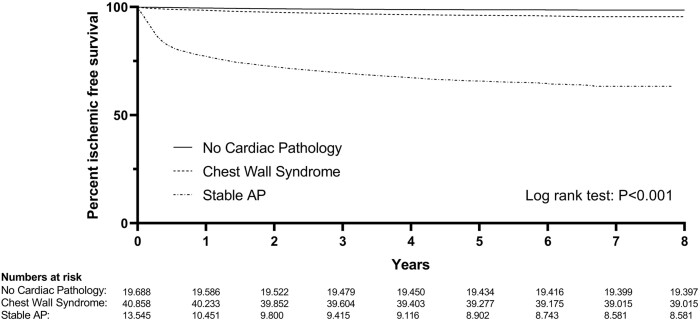
Kaplan–Meier ischaemic-free survival curve for 8 years of follow-up.

*Figure 3* shows a Kaplan–Meier plot of the patient with an ischaemic-free survival. After 1 year, 99% of the patient in the group with no cardiac pathology and chest wall syndrome, had an ischaemic-free survival. The 1-year ischaemic-free survival of the group with stable AP was 77%. The percentage of patients with an ischaemic-free survival at 5 and 8 years of follow-up included 99% and 98% in the group with no cardiac pathology, respectively. In the group with chest wall syndrome, the ischaemic-free survival was 96% at 5 years of follow-up and 95% at 8 years of follow-up. In the group with stable AP the ischaemic-free survival was lower, with 66% at 5 years and 63% at 8 years. There seemed to be a slightly lower chance of developing cardiac symptoms for the ‘no cardiac pathology’ when compared with the ‘chest wall syndrome’ group. This is surprising, since both groups are considered to not have any cardiac issues after discharge. It might be that cardiologists are more likely to give a ‘no cardiac pathology’ diagnosis when there is more clarity on the cause of the chest pain.

## Discussion

This study was conducted to gain insight into the diagnostic effort for patients with non-acute chest pain in the hospital. The findings of this study can be summarized as follows: (i) 82% of the patients referred to the hospital with suspicion of cardiac chest pain had a non-cardiac origin; (ii) a total of €142.7 million has been spent on 74 091 patients, of which €67 million was spent on non-cardiac patients; and (iii) after 8 years of follow-up, ≥95% of the patient diagnosed with no cardiac pathology or chest wall syndrome had an ischaemic-free survival. Data from one tertiary and three general hospitals were analysed. Two of the four hospitals were PCI centres. A total of 180 000 patients without a cardiac history are observed with chest pain per year in the Netherlands.^9^ In this study, 74 091 patients were included over a period of 5.7 years. This represents approximately 13 000 patients per year amounting to 7% of the national population. Patients with a cardiac history (18%, *Figure 1*) were excluded, this will lead to an underestimation of the total incidence of non-acute cardiac chest pain. These patients were excluded to facilitate interpretation of the data obtained, as patients have the same cardiac history (i.e. none). Compared with previous studies of chest pain in a non-acute setting baseline characteristics were comparable.^5^^,^^16^ Since the cohort can be considered representable for the national population, we believe that the findings in this study are representable too.

### The accuracy of patient referral

The current study showed a high incidence of non-cardiac chest pain in the referred patients (82%): 27% of the patients were diagnosed with no cardiac pathology and 55% of the patients were diagnosed with chest wall syndrome. As the patients were referred to the hospital with the suspicion of a cardiac cause, the ‘hit rate’ of an actual cardiac cause, for non-acute cases, seems low.

A study from Dumville *et al*.^17^ studied the long-term outcome of patients with chest pain who were referred from primary care towards a rapid access chest pain clinic in a non-acute setting. A total of 52% had non-cardiac chest pain after a follow-up of 6 months. Similar results were found in a study by Byrne *et al*.,^16^ where 633 patients were referred to a rapid access chest pain clinic. An incidence of low risk or non-cardiac chest pain in 51% of the patients was found after 8 months. In the current study, an incidence of 82% was found. The large difference can have several causes. For instance, the current study included patients based only on the initial diagnosis. In contrast, both other studies allow for a change in diagnosis during follow-up (i.e. from non-cardiac to cardiac). Furthermore, both studies from Dumville *et al*. and Byrne *et al*. were performed in the UK. It is possible that the country-specific circumstances with regard to referral to hospitals in the case of non-acute chest pain are different, resulting in a seemingly higher hit-rate of the British GP’s in the described situation.

### Costs and healthcare utilization

The total cost to obtain the diagnoses ‘no cardiac pathology’ was €13.5 million (€685 per patient). For ‘chest wall syndrome’, the total diagnostic expenditure was €55.5 million (€1360 per patient). A study from Mourad *et al*.^18^ analysed the extent of costs in secondary care, incurred by patients (*N* = 199) with non-cardiac chest pain and compared this with the costs of patients with acute myocardial infarction and AP. The annual cost per patient with non-cardiac chest pain was €10 068, which included also costs in primary care, indirect costs on productivity, loss due to sick-leave and medical costs. In the current study, only the direct costs (TD-ABC) were calculated per patient and could explain the lower costs found in the current study.

To extrapolate these costs to the total Dutch population, data from Dutch National Healthcare Institute is used.^9^ In this report, the annual amount of new patients with diagnostic codes 201 and 202 were reported to be *n* = 180 000. In this report, the diagnostic codes 201 (55%), 202 (18%), and 101 (27%) were included. To accurately compare the data, the mentioned 180 000 only represents 73% of the hypothetical national cohort as considered in this study. The total cohort would then be *n* = 247 000. The annual diagnostic cost for ‘no cardiac pathology’ is then €45.6 million, and the annual diagnostic cost for ‘chest wall syndrome’ is €184.4 million. The total annual expenditure to ascertain the absence of a cardiac cause for chest pain is then €230 million. We could call these ‘assurance costs’.

In the current study, referred patients were subjected to a wide variety of diagnostic tests. Furthermore, the utilization rate of non-invasive diagnostics was high in all groups: ≥90%. Hoorweg *et al*.^5^ investigated the utilization of diagnostic tests in an observational study, by including patients (*N* = 281) with acute and non-acute chest pain in primary care. A total of 44% of the patients underwent diagnostic testing. The higher utilization rate of the current study might be explained by the secondary care setting and the focus on non-acute chest pain: the clinical presentation might not be as clear as in an acute setting and therefore requires more diagnostic tests. In addition, the high utilization rate emphasizes the need to increase the compliance with the current guidelines. The European Society of Cardiology (ESC) guidelines recommend no non-invasive testing in patients with a pre-test probability (PTP) score of <15%.^19^^,^^20^ The guidelines do leave room for testing below 15% in particular when symptoms are limiting, but this should only apply to a small part of the cohort.^20^ The present study showed a diagnosis of cardiac chest pain in 18% of the total cohort. Under the assumption that the total incidence of cardiac chest pain of 18% implies an average PTP score of 18%, it is highly unlikely that more than 90% of the cohort had a PTP of more than 15%. Over investigation is a well-known problem and paralleled with high healthcare expenditures, hence an important factor in reducing healthcare costs.^21^

### Clinical outcome and follow-up

The current study showed that ≥95% of the patients in the group with no cardiac pathology and the group with chest wall syndrome had an ischaemic-free survival after 8 years. A total of 68% of the patient with stable AP had an ischaemic-free survival after 8 years. It can be concluded that the current system is very effective in distinguishing those that have an underlying cardiac cause for their chest pain and those that have not.

### Limitations

Some limitations should be acknowledged. First, this is an analysis based on a large-scale financial database. Previous studies showed a correlation between medical charts and financial data.^13^ However, in this study, this was not verified and might result in over- or under-diagnosis. For similar data, the Dutch National Healthcare Institute used a 15% extrapolation factor. Since TD-ABD costing is used in this study, there is no available data to come to a similar extrapolation factor. Since we do not use an extrapolation factor, we are confident our costing data is conservative. Secondly, there is currently no universal definition for non-acute chest pain. Depending on the doctor, a patient with non-cardiac chest pain can be diagnosed with ‘no cardiac pathology’ or with ‘chest wall syndrome’. For this reason, we defined non-acute suspected cardiac chest pain by including ‘no cardiac pathology’, ‘chest wall syndrome’, or ‘stable angina pectoris’. The diagnosis ‘no cardiac pathology’ can also be given to patients that are not referred due to chest pain. This might also be reflected in *Table 3*, where a different amount of diagnostic resources are used among the group with ‘no cardiac pathology’ compared with the groups with ‘chest wall syndrome’ and ‘stable AP’. The focus of this study was to investigate the diagnostic course of non-acute cardiac sounding chest pain, in the outpatient clinic. By careful selection of included diagnostic codes and exclusion of emergency department patients, we are confident that the vast majority of the cohort is from the outpatient clinic. Despite this focus, it is possible that patients were included who came with chest pain in an acute setting. Fourth, information about medication was not available in the database and has not been included in our analysis. This probably leads to an underestimation of the real costs. Fifth, this research analysed anonymous data from four different hospitals, in case a patient has been treated in multiple hospitals that patient could be included two or more times in the database. Finally, it is worth noting that the incidence of ECGs was not nearing 100%, which one would expect based on diagnostic guidelines.^20^^,^^22^^,^^23^ It is likely that not all ECGs were accounted for in the declaration database. In fact, it can be expected that an ECG was made in all patients.

### Future implications

The Dutch National Healthcare Institute showed that the current guidelines were not always followed in obtaining a diagnosis. This might be a reason for the large variety of applied diagnostics. This is also shown in the current study. The variety of applied diagnostics was large and most notably, the amount of performed exercise ECGs was large, even though the sensitivity of this test is quite low at 58% (95% CI 46–69%).^19^ The ESC and National Institute for Health and Care Excellence (NICE) guidelines clearly underline the importance of a careful history, i.e. a clear distinction in typical, atypical and non-specific chest pain in the diagnosis of cardiac sounding chest pain.^20^^,^^23^ The outcomes in the study by Sekhri *et al*.^24^ supported the distinctions (atypical, typical, and non-specific) in these guidelines. It is worthwhile to investigate why guidelines are frequently not followed, as the goal of these guidelines is to deliver effective and efficient healthcare.

The mentioned ‘assurance costs’ are currently considered a necessary expenditure. As discussed earlier, to stay ahead of the curve with regards to the rising healthcare costs, it follows that we should search for ways to drive the assurance costs down. One such way could be to switch to an integrated care model, which has shown promise in earlier research.^25^ Integrated care may play a large role in value based-healthcare; measuring costs in combination with health outcomes and patient experiences.^26^ Several previous studies correlated patient-level costs and outcome analyses to improvement in healthcare quality.^27^^,^^28^Nation-wide quality measurement and benchmarking feedback are already conducted in surgery.^28^ In future perspective and in the pursuit of improving cardiac care, extending the analysis of this study by benchmarking the hospitals on a nation-wide level, or benchmarking the performances of healthcare systems in countries can provide useful data.

Similar studies such as this study can be performed for other diagnostic courses where there is a high expenditure, and a desire to find out where the effort is spent.

Mourad *et al*.^18^ showed that patients with no cardiac chest pain use a significant amount of healthcare resources and costs society a substantial amount of money. To gain insight into the entire diagnostic course of non-acute chest pain and it’s corresponding costs, it would be interesting to investigate also the behaviour and potential recurrent referrals of patients who were diagnosed with non-cardiac chest pain.

## Conclusion

The prevalence of patients with non-acute chest pain is high and a significant amount of €142.7 million is spent on this particular patient group of 74 091 patients. This included a mean expenditure of €685, €1360, and €5483 per patient with no cardiac pathology (27% of cohort), chest wall syndrome (55%), or stable AP (18%), respectively. In the majority (82%) no cardiac cause of chest pain was found. Furthermore, ≥95% of the patients in the group with no cardiac pathology and chest wall syndrome did not develop an ischaemic event after 8 years of follow-up.

In conclusion, it is found that the Dutch health-care system is very effective in determining the underlying cardiac cause for non-acute chest pain. Extrapolating the data, it is also found that the diagnostic expenditure to ascertain non-cardiac chest pain is €230 million per year in the Netherlands. Not only is the monetary expenditure high, the time expenditure by the healthcare system is also high. In a time where the healthcare workers are under a permanently high workload, and where recent events have shown that the healthcare system needs extra capacity to deal with crises, we should define what we as society find acceptable as ‘assurance costs’.

## Supplementary material

Supplementary material is available at *European Heart Journal – Quality of Care and Clinical Outcomes* online.

## Supplementary Material

qcaa064_Supplementary_Data
