# Supplementary material for: Utilization of diagnostic resources and costs in patients with suspected cardiac chest pain
Source: Eur Heart J Qual Care Clin Outcomes. 2020 Aug 18;7(6):583–90. doi: 10.1093/ehjqcco/qcaa064 (PMC9172873; doi:10.1093/ehjqcco/qcaa064)
Supplement: qcaa064_Supplementary_Data [file qcaa064_supplementary_data.zip › Table 2 supplement.docx]

| **Category** | **Subcategory** | **CTG-code with description (Dutch)** |
| --- | --- | --- |
| Cardiac invasive Diagnostics or Treatment | Percutaneous Coronary Intervention (PCI) | \| 190343. Drug eluting cardiovasculaire stent (zie 190399 voor biologisch oplosbare cardiovasculaire stent. zie 190342 voor klassieke cardiovasculaire stent). \| \| --- \| \| 33231. PTCA eentak ter opheffing/verwijdering stenosen coronaire arterien. \| \| 33232. PTCA meertak of hoofdstam ter opheffing/verwijdering stenosen coronaire arterien. \| \| 33238. Acute PTCA ter opheffing/verwijdering stenosen coronaire. \| \| 33233. PTCA ter opheffing/verwijdering chronische occlusie coronaire arterien. \| \| 190342. Klassieke cardiovasculaire stent (zie 190343 voor drug eluting cardiovasculaire stent. zie 190399 voor biologische oplosbare cardiovasculair stent). \| \| 33234. PTCA met passage coronaire arterien graft. \| \| 190399. Biologisch oplosbare cardiovasculaire stent (zie 190343 voor drug eluting cardiovasculaire stent. zie 190342 voor klassieke cardiovasculaire stent). \| \| 190621. Drug Eluting ballon. \| |
|  | Coronary Angiography (CAG) | \| 33229. Catheterisatie linker en evt. rechter hart. inclusief evt. angiografie (coronair en/of hart en/of arteria pulmonalis). drukmeting. O2-bepaling (arterieel en/of hart). aortografie. aansl.uitw.pm. \| \| --- \| \| 33236. Intracoronair fysiologisch onderzoek. \| \| 33219. Catheterisatie van het rechter hart. inclusief eventuele drukmeting. O2-bepaling. angiografie (veneuze hartcatheterisatie). aansluiting van een uitwendige pacemaker. \| \| 33219. Catheterisatie van het rechter hart. inclusief eventuele drukmeting. O2-bepaling. angiografie (veneuze hartcatheterisatie). aansluiting van een uitwendige pacemaker. \| \| 80025. Al dan niet selectief onderzoek via percutane veneuze catheterisatie - alleen een venapunctie is inbegrepen in het desbetreffende codenummer  zoals b.v. 088012 en 084025. \| \| 80023. Al dan niet selectief onderzoek via percutane arteriële catheterisatie. \| \| 39530. Arteriografie. \| \| 85720. Radiologisch onderzoek arteriae coronariae. \| |
|  | ICD/PM | \| 190334. Biventriculaire ICD. \| \| --- \| \| 190329. Inwendige hartritmemonitor (ILR). \| \| 190623. Automatische implanteerbare cardioverter defibrillator (AICD) - 2 elektroden systeem. \| \| 33272. Het inbrengen van twee endocardiale elektroden en het aansluiten van een subcutaan geplaatste pacemaker. \| \| 33283. Vervanging van een pacemaker. \| \| 190331. 1 Draads pacemaker. \| \| 190622. Automatische implanteerbare cardioverter defibrillator (AICD) - 1 elektrode systeem. \| \| 33280. Het inbrengen van twee endocardiale elektroden en het aansluiten van een subcutane automatische defibrillator.  inclusief het aansluitend testen van de defibrillatiedrempel onder algehele anesthesie. \| \| 39698. Inbrengen van een subcutaan geplaatste hartritmemonitor (ILR). \| \| 33298. Implantatie van subcutane automatische defibrillator (AICD) excl. elektroden. incl. aansluiten elektroden en afregelen van defibrillator. \| \| 33267. Het inbrengen van een stimulatie-elektrode en het aansluiten van een subcutaan geplaatste pacemaker. \| \| 33291. Vervangen van subcutane automatische defibrillator (AICD) excl. elektroden. incl. aansluiten elektroden en afregelen van defibrillator. \| \| 190333. Biventriculaire pacemaker. \| \| 39699. Verwijderen van een subcutaan geplaatste hartritmemonitor (ILR). \| \| 33282. Het inbrengen van een of twee endocardiale elektroden plus sinuscoronarius elektrode en aansluiten subcut.automatische defibrillator.  incl. aansluitend testen van defibrillator onder alg.anesthesie. \| \| 33265. Atrial pacing + hisbundel-electrocardiografie + therapeutische uitwendige pacemaker. \| \| 33279. Het inbrengen van twee endocardiale elektroden en één sinus coronarius elektrode. en het aansluiten en het afregelen van een biventriculaire pacemaker. \| \| 33266. Het inbrengen van een stimulatie-elektrode en het aansluiten van een uitwendige pacemaker. \| \| 33284. Verwijdering van een pacemaker. \| \| 33262. Atrial pacing + hisbundel-electrocardiografie. \| \| 33286. Het bevestigen van een stimulatie-elektrode op het epicard na het openen van het hartzakje en het aansluiten van een subcutaan geplaatste pacemaker. \| \| 33287. Inbrengen endocard.elektrode en bevestigen tweede elektrode op het epicard. of bevestigen beide elektroden op epicard na openen hartzakje en aansluiten subc.pacemaker. \| \| 190609. Implanteerbare intracardiale pacemaker (transkatheter pacemaker). \| \| 33259. Inbrengen implanteerbare intracardiale pacemaker. \| \| 33260. Atrial pacing. \| |
|  | Valve Surgery | \| 190291. Aortaklepprothese. \| \| --- \| \| 33247. Percutane hartklep implantatie. \| \| 33250. Percutane hartklepplastiek. exclusief catheterdilatatie van hartklep (zie 033241). \| \| 33079. Hartklepvervanging. open procedure. \| \| 190666. Mitraclip. \| \| 33241. Catheterdilatatie van hartklep. \| \| 33078. Hartklepplastiek. open procedure. \| \| 33097. Vervanging aortaklep. aortawortel en aorta ascendens. \| \| 33152. Correcties aan de intra-thoracale grote vaten onder andere aortaruptuur. respectievelijk aneurysma arteria anonyma. \| \| 190292. Mitralisklepprothese. \| \| 33248. Transkatheter hartklepimplantatie. open procedure. \| \| 33295. Implanteren en aansluiten device voor korte termijn circulatoire ondersteuning hart. \| \| 190613. Intra-aortale ballon (endoclamp). \| \| 190624. Hartklepring. \| \| 190294. Tricuspidalisklepprothese. \| \| 190629. Klepdragende vaatprothese. \| \| 190619. Transkatheter hartklep. \| \| 190293. Pulmonalisklepprothese. \| |
|  | Other | \| 32602. Proefthoracotomie (zie 032606 voor proefthoracoscopie). \| \| --- \| \| 33170. Rethoracotomie met extracorporale circulatie tijdens dezelfde opname. \| \| 80828. Embolisatie van vaten. \| \| 32904. Pericardpunctie (zie 032900 voor open procedure en 032905 voor thoracoscopisch). \| \| 33242. Cathetersluiting CQ dilatatie van congenitaal of verworven hartvitium. \| \| 39482. Intracardiale echografie (ICE). \| \| 32672. Cardiothoracale operatie-unit standby. \| \| 32996. Ballonpomp per open procedure als bijkomende ingreep. \| \| 33043. Correctie van abnormale coronairverbindingen zoals fistels of een abberante oorsprong. \| \| 32992. Ballonpomp per punctie als zelfstandige ingreep. \| \| 39687. Intraveneuze trombolyse (IVT). \| \| 33040. Sluiten van een atrium-septum defect. type 2. \| \| 80028. Niet-electieve embolisatie van vaten. \| \| 32993. Ballonpomp per punctie als bijkomende ingreep. \| \| 33130. Operatie wegens een of meerdere tumoren van het atrium. onder andere myxomen. \| \| 32995. Ballonpomp per open procedure als zelfstandige ingreep. \| \| 33611. Intraveneuze trombolyse (IVT) bij acute diepe trombose (bij niet acute diepe trombose zie 033610). \| \| 39829. Intra-arteriële bloeddrukmeting. \| \| 32902. Subtotale pericardresectie. \| \| 190614. Accu VAD. \| \| 33029. Cardiomyectomie. \| \| 32900. Openen van het hartzakje zonder ingreep aan het hart. eventueel drainage van een pericarditis. via een thoracotomie. open procedure (zie 032904 voor percutaan en 032905 voor thoracoscopisch). \| \| 39096. Arteriepunctie door middel van een verblijfnaald voor registratie van de arteriële bloeddruk. \| \| 190620. Septal Occluding Device. \| \| 33049. Percutane mechanische linker hartoor sluiting. \| |
|  | FFR | \| 39476. Fractional Flow Reserve (FFR. voor Intracoronaire OCT zie 033256. voor IVUS zie 039477). \| \| --- \| \| 39477. IntraVasculaire UltraSound (IVUS. voor Intracoronaire OCT zie 033256. voor FFR zie 039746). \| |
|  | EFO/Ablatie | \| 32946. Catheterablatie linker atrium. \| \| --- \| \| 32944. Catheterablatie linker ventrikel. \| \| 32940. Hisbundel-catheterablatie. \| \| 32941. Catheterablatie rechter atrium. \| \| 32945. Catheterablatie rechter ventrikel. \| \| 32942. Catheterablatie accessoire bundel. \| \| 33076. MAZE-procedure. open. \| \| 33076. MAZE-procedure. open. \| \| 33077. Endoscopische MAZE-procedure. \| \| 33240. Alcoholablatie HOCM. \| \| 80058. RF-ablatie aandoening. \| |
|  | CABG | \| 33104. Aortocoronaire bypass operatie met uitsluitend veneuze graft(s) en/of kunststof materiaal. \| \| --- \| \| 33106. Aortocoronaire bypass operatie met 2 arteriële grafts. inclusief eventuele veneuze graft(s) en/of kunststof materiaal. \| \| 33105. Aortocoronaire bypass met 1 arteriële graft. inclusief eventuele veneuze graft(s) en/of kunststof materiaal. \| \| 33107. Aortocoronaire bypass operatie met 3 of meer arteriële grafts. inclusief eventuele veneuze graft(s) en/of kunststof materiaal. \| |
|  | Aortic Aneurysm | \| 33095. Vervanging aortawortel. aorta ascendens en aortaboog. \| \| --- \| \| 33152. Correcties aan de intra-thoracale grote vaten onder andere aortaruptuur. respectievelijk aneurysma arteria anonyma. \| \| 33096. Vervanging aortawortel. \| \| 33096. Vervanging aortawortel. \| \| 80930. Inbrengen van een aorta stentgraft in samenwerking met een chirurgisch team. \| \| 33094. Vervanging aortaboog. \| \| 33098. Vervanging aorta ascendens zonder circulatiestilstand. \| \| 33089. TAAA (thoraco-abdominaal aorta aneurysma). open procedure. \| \| 33093. Vervanging aorta ascendens met aortaboog. \| \| 190626. Klassieke aortabuisprothese onvertakt (zie 190627 met zijtak(ken).190628 voor endovasculaire aortabuisprothese). \| \| 33554. Reconstructie aan de aorta of haar directe zijtakken zoals arteria renales en arteria iliaca. open procedure (zie 033555 voor endovasculair). \| |
|  | LV restoration | \| 33110. Resectie aneurysma van de linkerventrikel. \| \| --- \| \| 33112. Sluiten van een ventrikel-septum perforatie. \| |
| Cardiac Non-Invasive Diagnostics | Laboratory | \| 79991. Ordertarief klinisch-chemische en microbiologische laboratoriumonderzoeken. inclusief bloedafname. \| \| --- \| \| 70402. Glucose Galactose-tolerantietest (i.v. belasting) Lactose-tolerantietest Maltose-tolerantietest Saccharose tolerantietest Tolbutamide tolerantietest. \| \| 70419. Kreatinine. \| \| 74899. Troponine. cardiale isovorm. \| \| 70702. Hemoglobine (incl. (eventueel) hematocriet en celindices (MCV. MCH en MCHC en erytrocyt)). \| \| 77121. Leucocyten. enkelvoudige bepaling. \| \| 70442. Natrium. \| \| 70715. Trombocyten tellen. \| \| 70689. C-reactive proteinen (CRP). \| \| 74891. ALAT. SGPT. Transaminase. \| \| 72649. Ferritine. \| \| 190430. Erytrocyten. \| \| 70820. BNP/NT-proBNP. \| \| 70489. ASAT. SGOT. transaminase. \| \| 74892. Melkzuur dehydrogenase (LDH). \| \| 70116. Ureum. \| \| 72417. Gamma-glutamyl-transpeptidase. \| \| 74895. Kreatine-fosfokinase. \| \| 74251. Cholesterol. HDL. \| \| 70703. Bezinkingssnelheid. \| \| 74896. Alkalische fosfatase. \| \| 72573. Thyrotrofine (TSH). \| \| 77434. Fibrine/fibrinogeen degradatie produkten. kwantitatief. \| \| 74110. Bilirubine. kwantitatief totaal of direct. elk. \| \| 72570. Thyroxine (vrij T4). \| \| 75044. Bloedkweek (aeroob + anaeroob). \| \| 70460. Triglyceriden. \| \| 70611. Antistoffen. vrije. tegen erytrocyten met behulp van poly-specifiek antiglobulineserum (indirecte Coombstest). \| \| 70718. Differentiele telling (machinaal). \| \| 70468. Melkzuur. \| \| 70425. Cholesterol. totaal. \| \| 75043. Kweekproef > 3 media. bacteriologisch. \| \| 70714. Eosinofielen tellen. \| \| 70424. Alkali reserve. \| \| 70404. Kreatinine klaring (of andere klaring). \| \| 72414. Bloedgassen: pH. pCO2. pO2 en/of standaardbicarbonaat van het arteriele bloed. \| \| 70707. Protrombinetijd. \| \| 72410. O2-verzadiging van het arteriele bloed. \| \| 70507. Resistentiebepaling kwantitatief m.b.v. MRC/Etest per antibioticum. bacteriologisch. \| \| 70004. DNA-amplificatie. kwalitatief. handmatig. \| \| 70307. Amylasebepaling - op alle materialen. exclusief fecaal materiaal (zie 070208). \| \| 71118. Antistoffen. IgT. IgG of IgA tegen elk micro-organisme m.b.v. immunoassay. \| \| 70604. Bloedgroep ABO + Rhesusfactor Rhesusfactor (D+ of D-) buisjestest. Rhesusfactor (D+ of D-) slide test of bromeline objectglas methode. \| \| 70706. Protrombinetijd bij orale antistolling. \| \| 77371. APTT. geactiveerde partiele tromboplastinetijd. \| \| 72826. Koolmonoxide. kwantitatief met identificatie in bloed. \| \| 70119. Chloride. \| \| 76875. Kruisproef. volledig (3 methoden). \| \| 190449. Trombocyten. aferese. bestraald. 1/4 gesplitst. \| \| 74065. HbAlc. \| \| 190460. Plasma. aferese. \| \| 75045. Determinatie micro-organismen. bacteriologisch. \| \| 70426. Calcium. kwantitatief in ander materiaal dan faeces. \| \| 75042. Kweekproef 2 - 3 media. bacteriologisch. \| \| 190489. Overige kort-houdbare bloedproducten \| \| 72820. Alcohol. kwantitatief met identificatie. \| \| 74802. Albumine. \| \| 50512. Complexe moleculaire diagnostiek - bepalingen op geïsoleerd DNA. RNA of eiwit anders dan freq.aangevr.bepalingen op micro-organismen (excl.mammaprint 050530. oncotype DX 050531 of BRCA1-like 050532). \| \| 70407. Methemoglobine. sulfhemoglobine. elk. \| \| 70469. Magnesium. \| \| 74801. Eiwitbepaling - op alle materialen. exclusief fecaal materiaal (zie 070211). \| \| 70100. Urine screening kwalitatief zonder sediment Aceton Bilirubine Eiwit. kwalitatief Glucose. kwalitatief Reactie Soortelijk gewicht. \| \| 190440. Trombocyten samengevoegd. \| \| 70197. Metanefrinen. normetanefrinen. \| \| 71137. Microbieel antigeen of toxine direct in patiëntenmateriaal m.b.v. bijvoorbeeld immunoassay. bacteriologisch. \| \| 71105. HBs antigeen. \| \| 50503. Immuno-pathologisch onderzoek. \| \| 70005. RNA-amplificatie. kwalitatief. \| \| 70501. Microscopisch onderzoek (ongekleurd of gekleurd). elk. \| \| 77091. Lymfocyten subpopulatie. eerste antistof. \| \| 71126. Antistoffen. IgM tegen elk micro-organisme m.b.v. immunoassay. \| \| 70003. DNA-amplificatie. kwalitatief. geautomatiseerd. \| \| 72886. Cardiaca. identificatie en/of kwantificatie. m.b.v. chromatografie. \| \| 70487. Fibrinogeen. \| \| 50501. Pathologisch-anatomisch (histologisch) onderzoek en overige cytodiagnostische onderzoeken. \| \| 70303. Sediment. \| \| 72621. Prostaatspecifiek antigeen (PSA). \| \| 74897. CK-MB. kreatine-fosfokinase iso-enzym. \| \| 71144. Microbieel antigeen of toxine direct in patiëntenmateriaal m.b.v. bijvoorbeeld immunoassay (m.u.v. HBs ag). infectieserologisch. \| \| 70001. Hybridisatie. geautomatiseerd. \| \| 70440. Vitamine D (dihydroxycholecalciferol). \| \| 72583. Vitamine B12. cyanocobalamine. \| \| 50518. Naaldbiopt. complexe cytologische punctie. \| \| 79010. Bloedgroep erytrocytenserologie. bijzonder (CLB-B). \| \| 70421. Fosfaat. \| \| 70215. Melkzuur. kwantitatief. \| \| 72582. Foliumzuur. \| \| 70717. Differentiele telling (hand). \| \| 70212. Calcium in faeces. \| \| 70123. Homocysteine. \| \| 70449. Transferrine. \| \| 79011. Bloedgroep erytrocytenserologie. bijzonder (CLB-C). \| \| 50514. Eenvoudige moleculaire diagnostiek - bepalingen op coupes weefsel en/of cellen (oa CISH. ISH. FISH) en frequent aangevr.bepalingen op aanw.andere micro-organismen dan HPV (o.a.TBC.EBV.HSV.Bartonella). \| \| 70716. Reticulocyten tellen. \| \| 70006. DNA/RNA-amplificatie. kwantitatief. \| \| 74151. Lipasebepaling - op alle materialen. exclusief fecaal materiaal (zie 070209). \| \| 190470. Omniplasma. \| \| 70614. CDE fenotypering (rhesusfactor. subtypering). \| \| 70505. Resistentiebepaling kwalitatief m.b.v. diffusie-methode. bacteriologisch. \| \| 74336. Immuno-electroforese met antiserum. inclusief eventuele determinatie. \| \| 79005. Auto-immuunziekten. overige. bijzonder (CLB-C). \| \| 71141. Microbieel antigeen of toxine direct in patiëntenmateriaal m.b.v. bijvoorbeeld immunoassay. virologisch. \| \| 70496. Osmolariteit. \| \| 72424. Apolipoproteine A. B. C. E. \| \| 72866. Immunomodulantia / immunosuppressiva m.b.v. chromatografie. \| \| 70437. IJzer. \| \| 77291. Haptoglobine. \| \| 71511. Vitamine B1. Thiamine. \| \| 70007. DNA/RNA-analyse (bv. sequentie-bepaling of sub-typering). \| \| 72554. Thyrotrofine (TSH) binding inhibitor. \| \| 71471. Hydroxy-indolazijnzuur. 5-. kwantitatief. \| \| 72893. Antimicrobiele middelen. m.b.v. immunoassay. \| \| 72604. Bloedgroep bepalingen niet vallende onder ABO. rhesus (Duffy. Kell. etc.) per bloedgroep. \| \| 79006. Bloedcelchemie. rood. bijzonder (CLB-B). \| \| 70110. Indican. \| \| 71122. Antistoftiterstijging met behulp van neutralisatie (2 bepalingen). \| \| 72571. Trijoodthyronine (vrij T3). \| \| 75041. Kweekproef < 2 media. bacteriologisch. \| \| 72894. Antimicrobiele middelen. m.b.v. chromatografie. \| \| 50505. Electronenmicroscopisch onderzoek. \| \| 72905. Toxicologisch onderzoek (algemeen) ter vaststelling of uitsluiting van intoxicatie. m.b.v. een chromatografische techniek en hierbij de kwantificatie van de hoofdcomponent. \| \| 50521. Complexe resectie. \| \| 70588. Microscopisch onderzoek op tuberculose (Ziehl-Neelsen of gelijkwaardige methode). \| \| 190432. Erytrocyten bestraald. \| \| 50510. Flow-cytometrie. \| \| 71739. Micro-albumine in urine. \| \| 75054. Determinatie micro-organismen. mycologisch. \| \| 70185. Antistoffen tegen elk micro-organisme m.b.v. immunoblot. \| \| 74335. Electroforetisch diagram in diverse media. eventueel met speciale kleuringen. met (relatief) kwantitatieve bepaling der fracties. eventueel inclusief totaal eiwitbepaling. \| \| 70610. Antistoffen. gebonden. tegen erytrocyten met behulp van poly-specifiek antiglobulineserum (directe Coombstest). \| \| 71512. Vitamine B6. Pyridoxine. \| \| 50516. Eenvoudig biopt. eenvoudige cytologie (excl. bepalingen op de aanwezigheid van micro-organismen (zie 050513 of 050514). \| \| 72106. Bloed. kwalitatief (tablet test). \| \| 72601. Antistoffen tegen specifiek humaan weefsel. \| \| 70693. Anti-nucleaire factor (ANF). \| \| 71125. Antistoffen. IgT. IgG of IgA tegen elk micro-organisme m.b.v. IF. \| \| 74769. Lambda ketens. vrij of gebonden. elk. \| \| 70130. Urinezuur. \| \| 72812. Opiumwet. screening middel vallende onder de Opiumwet m.b.v. immunoassay. per component tot max. 3 componenten per dag (zie ook 072813). \| \| 74767. Kappa ketens. vrij of gebonden. elk. \| \| 70108. Diaceetzuur. \| \| 71124. Antistoffen. IgM tegen elk micro-organisme m.b.v. IF. \| \| 70503. Kweekproef op tuberculose. \| \| 79018. Immunocytologie onderzoeken. bijzonder (CLB-C). \| \| 70446. Aminozurenchromatogram. \| \| 72575. Thyroxine (T4). \| \| 70476. Immunoglobuline. elk. \| \| 79001. Allergie-onderzoeken. bijzonder (CLB-B/CLB-C). \| \| 72840. Anti-epileptica. m.b.v immunoassay. elk. \| \| 74962. Colloid osmolaliteit (colloid osmotische druk). \| \| 72857. Lood. kwantitatief m.b.v. AAS in bloed. \| \| 79024. Leucocyten-/Trombocytenserologie. bijzonder (CLB-C). \| \| 79004. Reumafactoren (Elisa) (CLB-B). \| \| 70202. Bilirubine. kwalitatief. \| \| 79013. HLA-overige bijzondere onderzoeken (CLB-C). \| \| 70483. Ammoniak. \| \| 72620. Prostaat zure fosfatase (antigeen). \| \| 71143. Microbieel antigeen of toxine direct in patiëntenmateriaal m.b.v. bijvoorbeeld immunoassay. parasitologisch. \| \| 78041. Zwangerschapsreactie uit serum. \| \| 79003. Antistoffen tegen weefselantigenen. bijzonder (CLB-B). \| \| 72576. Trijoodthyronine (T3). \| \| 72646. Parathormoon (PTH). \| \| 71971. Vanillyl-amandelzuur (VMA). \| \| 70517. Beta lactamase test. \| \| 72420. IgG subklassen. \| \| 72602. Allergenen. (Specifiek IgE Antistof tegen. RAST). \| \| 72501. Cortisol. \| \| 72603. Allergenen. screening op inhalatie-allergie. \| \| 70475. Vetzuren. vrij (FFA. NEFA). \| \| 72630. Carcino-embryonaal antigeen (CEA). \| \| 70914. Protozoaire cysten (concentratie). \| \| 72559. hCG. betavrij-humaan choriongonadotrofine. \| \| 70901. Microscopisch onderzoek op parasieten (uitstrijkje. dikke druppel. eosine. jodium. elk). \| \| 71732. Electroforetisch diagram. na concentratie. in diverse media. eventueel met speciale kleuringen. met (relatief) kwantitatieve bepaling der fracties. eventueel inclusief totaal eiwitbepaling. \| \| 75053. Kweekproef > 3 media. mycologisch. \| \| 72648. DNA-antistoffen (kwantitatief). \| \| 70697. Cellulaire immuniteit door middel van lymfocyten transformatie. bepaling van. \| \| 70728. Hemoglobine scheiding kwantitatief. \| \| 79009. Bloedstollingsfactoren. bijzonder (CLB-C). \| \| 70913. Wormeieren (concentratie). \| \| 72557. hCG. humaan choriongonadotrofine. intact molecuul. \| \| 72558. hCG. beta-humaan choriongonadotrofine. \| \| 72809. Antidepressiva. identificatie en/of kwantificatie. \| \| 70428. Angiotensine converting enzym. \| \| 79015. Immunochemische onderzoeken. bijzonder (CLB-B). \| \| 50506. Verrichtingen van een punctie ten behoeve van cytologisch onderzoek. \| \| 72508. Aldosteron. \| \| 71120. Antistoffen tegen elk micro-organisme m.b.v. CBR of HAR per bepaling. \| \| 70710. Microscopie van sternumpunctaat. standaardkleuring en beoordeling. \| \| 74721. Koper. \| \| 71972. Homovanilline (HVA). \| \| 72555. Thyrotrofine (TSH) stimulating immuunglobuline. \| \| 70755. Circulerend anticoagulans (lupus anticoagulans. antitromboplastine. antifosfolipiden). per anticoagulans. \| \| 77102. Microscopie van punctaten. aanvullende specifieke kleuring: esterase. zure fosfatase. tartraat geremde zure fosfatase. sudan black. etc. inclusief beoordeling. \| \| 72887. Cardiaca. identificatie en/of kwantificatie. m.b.v. immunoassay. \| \| 70445. IJzerbindingscapaciteit. \| \| 77451. Antitrombine III activiteit. \| \| 75052. Kweekproef 2 - 3 media. mycologisch. \| \| 70526. Resistentiebepaling kwantitatief d.m.v. MRC/Etest per antibioticum. mycologisch. \| \| 75051. Kweekproef < 2 media. mycologisch. \| \| 74763. Kwantitatieve bepaling van een immunoglobuline. nefelometrisch. \| \| 72647. Renine. \| \| 72623. Carcinoom Antigeen (CA). elk. \| \| 70481. Cellen tellen in liquor. \| \| 72109. Hydroxyproline. \| \| 72543. C-peptide. \| \| 74804. Glycoproteine. alfa-I-zure. \| \| 77581. Proteine S totaal antigeen. \| \| 70601. Agglutinatiereactie volgens Widal. voor elk micro-organisme. \| \| 79993. CLB-referentietarief. \| \| 72884. Antipsychotica (neuroleptica). identificatie en/of kwantificatie. \| \| 72896. Lithium. \| \| 72900. Toxicologisch onderzoek (algemeen) ter vaststelling of uitsluiting van intoxicatie. voorproeven (kleurproeven. immunoassays waaronder paracetamol). \| \| 70734. Bloedstollingsfactor VIII. \| \| 72511. Testosteron. \| \| 79989. Ordertarief klinisch-chemische en microbiologische laboratorium bloedonderzoeken. exclusief bloedafname. \| \| 50502. Cytodiagnostisch onderzoek van een cervix-preparaat (deze code is niet bestemd voor onderzoek ten gevolge van het bevolkingsonderzoek). \| \| 77571. Proteine C activiteit. \| \| 70656. Complement component (kwantitatieve bepaling). \| \| 72502. Cortisol. vrij. \| \| 74805. Antitrypsine. alfa-I. \| \| 72551. Luteiniserend hormoon (LH). \| \| 72112. Oxaalzuur. \| \| 78252. Acetylglucoseaminidase. N. \| \| 72550. ACTH. corticotrofine. \| \| 70201. Bloed (occult). kwalitatief. \| \| 74901. Ceruloplasmine. \| \| 72888. Methotrexaat met immunoassay. inclusief eventuele herbepalingen. \| \| 72845. Analgetica/antirheumatica. m.b.v. immunoassay. \| \| 70455. Cholinesterase. \| \| 70222. Galzure zouten in faeces. \| \| 70704. Bloedingstijd. \| \| 72897. Theofylline. m.b.v. immunoassay. \| \| 72552. Follikelstimulerend hormoon (FSH). \| \| 79016. Immunochemische onderzoeken. bijzonder (CLB-C). \| \| 72903. Toxicologisch onderzoek (algemeen) ter vaststelling of uitsluiting van intoxicatie. m.b.v. een chromatografische techniek. \| \| 70917. Schistosoma (zoutzuur-ether concentratie). \| \| 72834. Metalen (zwaar) kwalitatief en/of kwantitatief per element (uitgezonderd lood) met vlamloze AAS. \| \| 71102. Typering van geisoleerd virusstam. \| \| 72811. Opiumwet. identificatie middel vallende onder de Opiumwet. chromatografisch. per component. \| \| 70524. Microscopisch onderzoek (gekleurd of ongekleurd). elk. mycologisch. \| \| 39114. Cytologische en/of bacteriologische punctie. \| \| 72841. Anti-epileptica. m.b.v. chromatografie. elk. \| \| 70211. Eiwitbepaling - fecaal materiaal (eiwitbepaling op andere materialen. zie 074801). \| \| 72631. Alfa-foetoproteine (AFP). \| \| 70417. Vitamine C. \| \| 70494. Cryoglobuline. kwalitatief. \| \| 70620. L-agglutinatie. \| \| 72813. Opiumwet. screening op middelen vallende onder de Opiumwet m.b.v. immunoassay. per pakket bestaande uit 4-8 componenten. \| \| 72519. Sex hormone binding globulin (SHBG). \| \| 72850. Nicotine/cotinine. chromatografisch. \| \| 70213. Vet (vetten. vetzuren. droge stof). kwantitatief. \| \| 70151. Vitamine B2. \| \| 70292. Porfyrines. kwantitatief. \| \| 72815. Laxantia. screening. \| \| 70126. Porfyrines. uro-. copro-. proto-kwantitatief. elk. \| \| 72531. Oestron. oestradiol. elk. \| \| 74064. Geglyceerde hemoglobine. \| \| 70219. Leucocyten in faeces. \| \| 72805. Benzodiazepinen. identificatie en/of kwantificatie. \| \| 72561. Groeihormoon. hGH. Somatropine. \| \| 70626. Treponema pallidum haemagglutinatietest (TPHA). \| \| 72565. Prolactine (PRL). \| \| 72803. Slaapmiddelen. identificatie en/of kwantificatie. \| \| 77094. HLA-B27. \| \| 70824. Bilirubine in vruchtwater. eventueel ascitesvocht. kwantitatief. \| \| 72640. Beta-2-microglobuline. \| \| 71012. Virologisch onderzoek door middel van celkweek 2 - 3 media. \| \| 72516. Dehydro-epi-androsteronsulfaat (DHEA-S). \| \| 74803. Myoglobine. \| \| 70450. Koolhydraat Deficïent Transferrine (CDT). \| \| 72542. Insuline-antistoffen. \| \| 70608. Paul en Bunnell. reactie van. \| \| 70628. VDRL-reactie (kwantitatief). \| \| 74058. Fructosamine. \| \| 70627. Fluorescerende treponemale anti-stoffenreactie (met toepassing van absorptie) FTA-ABS-reactie. \| \| 70827. Zink. \| \| 72541. Insuline. \| \| 70618. Anti-sterptolysine titer / anti-DNase B titer of stapholysine titer. elk. \| \| 70216. Onderzoek naar Calprotectine in feces. \| \| 72641. Calcitonine. \| \| 72814. Opiumwet. kwantificatie middel vallende onder de Opiumwet. chromatografisch. \| \| 70732. Bloedstollingsfactor V. \| \| 70733. Bloedstollingsfactor VII. \| \| 70220. Osmolariteit in faeces. \| \| 70114. Aminolevulinezuur. delta-. kwantitatief. \| \| 72422. Viscositeit. \| \| 72421. CI-esteraseremmeractiviteit. \| \| 72423. Precipitinen. \| \| 70761. Heparine bepaling (anti Xa activiteit). \| \| 70616. Agglutinatie. koude. \| \| 74758. Bence Jones eiwit. \| \| 72110. Myoglobine. kwalitatief. \| \| 70642. Precipitatie reactie. \| \| 72520. Somatomedine. \| \| 72816. Diuretica. screening. \| \| 70206. Vertering. kwalitatief. \| \| 70312. Bloed (occult) in diverse materialen. \| \| 71142. Microbieel antigeen of toxine direct in patiëntenmateriaal m.b.v. bijvoorbeeld immunoassay. mycologisch. \| \| 70741. Leucocytenconcentratie met kleuring. \| \| 70439. Lipoiden. totaal. \| \| 190450. Trombocyten. aferese. bestraald. 1/2 gesplitst. \| \| 79020. Klinische viro immunologie. bijzonder (CLB-C). \| \| 70525. Resistentiebepaling kwalitatief m.b.v. diffusie-methode. mycologisch \| \| 50509. Cytodiagnostisch onderzoek cervix-preparaat ivm bevolkingsonderzoek (preventief planmatig georganiseerd cytodiagnostisch onderzoek. waarbij het cervix-preparaat wordt afgenomen door de huisarts). \| \| 50517. Biopt. matig complexe cytologie. \| \| 50520. Complex biopt. matig complexe resectie. \| |
|  | Nuclear/SPECT | \| 120246. SPECT van hartkamers ECG-getriggerd met EF-berekening. inspanning en stress-test (244). \| \| --- \| \| 120245. SPECT van hartkamers ECG-getriggerd. rust met EF- berekening (244). \| \| 120241. SPECT van myocard inspanning met stress-test. \| \| 120244. SPECT van myocard vitaliteit. \| \| 120043. Ejectiefractie L.V. met wandbewegingsanalyse. \| \| 120240. SPECT van myocard rust. \| \| 120042. Ejectiefractie L.V. en/of R.V. met wandbewegingsanalyse. \| \| 120044. Ejectiefractie L.V. en R.V. met wandbewegingsanalyse. \| \| 39759. Bespreking nucleair cardiologisch onderzoek. \| |
|  | Ultrasound | \| 39494. Echografie van het hart. \| \| --- \| \| 39493. Oesophagus echocardiografie TEE. \| \| 39495. Dobutamine stress-echo. \| \| 85070. Echografie van het hart en/of de thorax. \| \| 120046. Cardiale shuntmeting. \| |
|  | Exercise test | \| 39844. Eenvoudige (fiets)ergometrie met opklimmende belasting met ECG-apparatuur en oscilloscoop tijdens een afzonderlijke afspraak. \| \| --- \| \| 39845. Uitgebreide (fiets)ergometrie met opklimmende belasting met ECG-apparatuur en oscilloscoop (incl. ventilatiemeting en O2 saturatie). \| |
|  | ECG | 39757. Beoordeling ECG. Holter. inspanningsonderzoek e.d. |
|  | Cardiac CT | \| 85042. CT onderzoek van de thorax. het hart en grote vaten inclusief inbrengen contrastmiddel. \| \| --- \| \| 85141. Multislice CT-hart tbv Ca2+-bepaling inclusief voor- en nabespreking met cardioloog. \| \| 85140. Multislice CT-hart inclusief voor- en nabespreking met cardioloog. \| \| 120560. CT onderzoek alleen voorafgaand aan PET of SPECT. \| \| 39496. Begeleiding en interpretatie door cardioloog bij multislice CT-hart inclusief voor- en nabespreking met radioloog. \| \| 39497. Begeleiding en interpretatie door cardioloog bij multislice CT-hart voor Ca2+-meting inclusief voor- en nabespreking met radioloog. \| |
|  | MRI | \| 85191. MRI-hart met dobutamine stress-test. \| \| --- \| \| 85190. MRI-hart. \| \| 39506. Begeleiding en interpretatie MRI-hart door cardioloog inclusief voor- en nabespreking met radioloog. \| \| 39507. Begeleiding en interpretatie dobutamine stress-test door cardioloog bij MRI-hart inclusief voor- en nabespreking met radioloog. \| |
|  | X-ray | \| 85002. Radiologisch onderzoek thorax. een of meerdere richtingen. inclusief doorlichting. \| \| --- \| \| 85000. Radiologisch onderzoek thorax. doorlichting zonder opname. \| \| 85120. Laevocardiografie. \| |
|  | Holter | 39755. Analyse van een 24-uurs electrocardiografie registratie. |
|  | Rhythm monitoring | \| 39843. Bewakingstelemetrie. \| \| --- \| |
|  | Other | 39848. 24-uurs bloeddrukmeting. |
| Emergency Department | General | \| 190218. Verpleegdag. \| \| --- \| \| 190013. Herhaal-polikliniekbezoek. \| \| 190060. Eerste polikliniekbezoek. \| \| 190011. Eerste polikliniekbezoek. \| \| 190012. Polikliniekbezoek. niet zijnde een eerste polikliniekbezoek. dat leidt tot opening van een nieuwe DBC. \| \| 190015. Spoedeisende hulp contact op de SEH afdeling. \| \| 39696. Preassessment. \| \| 39680. Perifeer infuus inbrengen. \| \| 39939. Aerosolbehandeling. \| \| 39830. Het inbrengen van een verblijfnaald ter verkrijging van arterieel bloed voor onderzoek naar pO2. pH en pCO2 voor en tijdens inademen van zuurstof. \| \| 39831. Het inbrengen van een verblijfnaald ter verkrijging van arterieel bloed voor onderzoek naar pO2 en pCO2 in rust en tijdens inspanning. zonodig ook na inademen van zuurstof. \| \| 39677. Life support S.E.H. \| \| 39604. Inbrengen arteriële lijn. \| \| 190016. Spoedeisende hulp contact buiten de SEH afdeling. elders in het ziekenhuis. \| \| 39676. ATLS traumaopvang - diagnosticeren en stabiliseren van verschillende typen (organische) letsels en/of perforaties onder verantwoordelijkheid van een ATLS gecertificeerd medisch specialist. \| \| 39852. Acute non-invasieve beademing op afdeling. \| |
|  | Invasive/surgery general | \| 33290. Behandeling met de cardioverter. \| \| --- \| \| 39881. Urologische behandeling dilataties en catheterisaties. \| \| 39090. Algehele anesthesie bij specialistisch onderzoek en bij verrichtingen. waarvoor geen anesthesie staat vermeld. \| \| 39829. Intra-arteriële bloeddrukmeting. \| \| 32684. Diagnostische pleurapunctie. \| \| 33289. Resuscitatie. met intubatie. al of niet met defibrillatie (zie 033288 voor zonder intubatie). \| \| 39941. Tracheaspoeling en/of maagheveling. \| \| 39445. Injecties. met uitzondering van injecties ten behoeve van locale anesthesie. \| \| 33288. Resuscitatie. zonder intubatie. al of niet met defibrillatie (excl. met intubatie zie 033289). \| \| 39096. Arteriepunctie door middel van een verblijfnaald voor registratie van de arteriële bloeddruk. \| \| 38644. Gesloten repositie bimalleolaire fractuur. \| \| 32688. Therapeutische pleurapunctie. \| \| 38651. Conservatieve behandeling bandlaesie van de enkel. \| |
| Inpatient Care | CCU | \| 190218. Verpleegdag. \| \| --- \| \| 190204. Verpleegdag. \| |
|  | Consult | \| 190013. Herhaal-polikliniekbezoek. \| \| --- \| \| 190060. Eerste polikliniekbezoek. \| \| 39679. Hartteambespreking. \| \| 192850. Intramurale diëtetiek (per kwartier). \| \| 190009. Klinisch intercollegiaal consult. \| \| 198202. Behandelcontact verpleegkundig specialist. \| \| 198202. Behandelcontact verpleegkundig specialist. \| \| 190017. Medebehandeling. \| \| 190005. Multidisciplinair overleg (MDO). \| \| 194164. Psychotherapiecontact - medisch psycholoog. \| \| 194162. Intake face-to-face contact - medisch psycholoog. \| \| 194165. Behandeling groepscontact - medisch psycholoog. \| \| 194156. Rapportage (brief. correspondentie. rapport) - medisch psycholoog. \| \| 194155. Gegevensbeheer (dossier. info. interpretaties. decursus)  - medisch psycholoog. \| \| 194163. Behandelingscontact - medisch psycholoog. \| \| 194160. Psychodiagnostisch - neuropsychologisch onderzoek - tests - medisch psycholoog. \| \| 190129. IC consult. Intercollegiaal consult buiten de IC. spoed en niet-spoed. \| \| 192844. Diëtetiek als onderdeel van gecoördineerde multidisciplinaire zorgverlening aan patiënten met DM. COPD en VRM (per kwartier.) \| \| 194157. Psychodiagnostisch - screening - tests - medisch psycholoog. \| \| 120412. Consult. niet gevolgd door een nucleair geneeskundige behandeling. \| \| 193022. Eenmalig onderzoek (logopedie). \| \| 190019. Screen to screen beeldcontact ter vervanging van een fysiek herhaalconsult (voor andere vormen van teleconsult zie 190025). \| \| 39230. Transplantatieteambespreking. \| \| 190854. Intercollegiaal consult arts - revalidatie. \| \| 194172. Consultaties - multidisciplinair overleg - medisch psycholoog. \| \| 190010. Multidisciplinair consult. \| \| 190022. Analyse behandeladvies en/of behandeling elders opgesteld en/of uitgevoerd. in het kader van een second opinion. \| \| 39580. Longteambespreking. \| \| 70027. Doelgerichte consultatie van ondersteunend specialist door poortspecialist bij al geopende DBC ivm direct patiënt gerelateerde vraagstelling. telefonisch of face-to-face. zonder aanwezigheid patiënt. \| |
|  | Daycare | \| 190035. Dagverpleging I. \| \| --- \| \| 190091. Langdurige observatie zonder overnachting. \| \| 190090. Dagverpleging. \| \| 190055. Dagverpleging II. \| \| 39691. Preassessment dagopname. \| |
|  | ICU | \| 190155. IC-dag zwaar. \| \| --- \| \| 190141. IC behandeldag groep 3. Een kalenderdag waarop op enig moment sprake is geweest van medische behandeling van een patiënt op de IC. \| \| 190153. IC-dag licht. \| \| 190154. IC-dag middel. \| \| 190142. IC opnametoeslag groep 3. Wordt geregistreerd op de eerste IC behandeldag. \| \| 190143. IC beademingstoeslag groep 3 \| \| 39610. Extra Corporele Membraan Oxygenatie (ECMO) bij behandeling van volwassenen (zie 039611 bij behandeling op Neonatale IC of Pediatrische IC). \| \| 190131. Interklinisch IC transport(>= 2 uur). Door medisch specialist begeleid transport van een IC-patiënt tussen ziekenhuizen. \| \| 39671. Acute beademing (IC) noninvasief. \| \| 190610. Device voor korte termijn circulatoire ondersteuning hart (o.a. Centrimag. ECMO. Impella. T-PLS etc.). \| \| 39672. IC medebehandeling (voor niet-IC medebehandeling zie 190017). \| |
|  | Function test general | \| 39778. Uitbreiding Trans Cranieel Doppler-onderzoek (TCD)/duplex met 1 aanvullende test. \| \| --- \| \| 33694. Doppler met eventuele polsvolumerecording (PVR). \| \| 39839. Spirografische longfunctiebepaling. \| \| 39832. Bepaling van de alveolaire ventilatie en analyse van de ongelijkmatigheid van de ventilatie. Katapherometrie en capnografie vallen onder deze code. \| \| 39932. Spirografisch onderzoek naar de invloed van bronchusverwijdende geneesmiddelen per injectie of per inhalatie. \| \| 39837. Residubepaling longen. \| \| 120060. Longperfusieonderzoek. \| \| 120061. Longventilatieonderzoek met edelgassen of aerosolen. \| \| 39774. Duplex extracraniële halsvaten. \| \| 39846. Beoordeling longfunctieonderzoek voor derden. \| \| 34686. Diagnostische endoscopie van het colon met behulp van flexibele endoscoop. eventueel inclusief biopten. poliepectomie of colonmanometrie. \| \| 39775. Duplex bloedvaten in extremiteiten. \| \| 39737. Onderzoek arteriële obstructies extremiteiten dmv bloeddrukmeting armen en/of benen of penis met CW doppler of plethysmografie incl PVR curven of doppler stroomsnelheid curven incl een belastingproef. \| \| 32480. Diagnostische bronchoscopie. inclusief een of meerdere proefexcisies. curettage en/of afzuigen van materiaal voor cytologisch en/of pathologisch onderzoek. \| \| 39836. Overzichtsbepaling van de mechanische ventilatieverhouding door middel van direct geregistreerde drukvolumediagrammen of luchtweerstandbepaling. \| \| 34391. Oesofagusfunctieonderzoek. inclusief eventuele 24-uurs metingen (manometrie. impedantiemetrie en/of pH-meting). \| \| 39938. Spirometrie. voor en na inspanning. \| \| 39738. Onderzoek veneuze afwijkingen extremiteiten dmv registreren veneuze CW dopplersignalen incl. proximale en distale compressietests en/of vasalva manoeuvres en/of outflow- of fotoplethysmografie. \| \| 39735. Polysomnografie (PSG) \| \| 39743. Standaard electromyografisch onderzoek (EMG) (< 45 min.). \| \| 120179. C14-ureumademtest. \| \| 39717. Aanvullende videoregistratie (tijdens EEG tot 1 uur). \| \| 39702. Standaard electro-encephalografie (EEG). registratie tot 1 uur. \| \| 39942. Huidreactie volgens Mantoux. \| \| 39818. Statische perimetrie. \| \| 39771. Standaard Trans Cranieel Doppler-onderzoek (TCD). \| \| 39933. Histamine (acethylcholine) provocatietest. Spirografisch onderzoek ter bepaling van de prikkelbaarheidsdrempel van de luchtwegen door middel van inhalatie-provocatietest(s). \| \| 39822. Biometrie oogbol. \| \| 39729. Slaap-Apneu registratie (screening). \| \| 39601. Onderzoek gevoeligheid allergenen dmv huidtest(s). dmv huidpriktests of intracutane injecties. \| \| 39833. Bepaling van de weerstand van de luchtwegen en longparenchym afzonderlijk + bepaling van de volledige elasticiteitskarakteristiek van de longen. \| \| 37532. Cardiotocografie. \| \| 190702. Toonaudiometrie - audiologisch centrum. \| \| 39794. Eenvoudige toon-audiometrie. \| \| 39900. Electroshock. per behandeling. \| \| 39344. Duodenaalsondage. inclusief pancreas functie onderzoek. \| \| 39581. Beperkt CGA in het kader van een medebehandeling. \| |
|  | General | \| 190218. Verpleegdag. \| \| --- \| \| 190204. Verpleegdag. \| \| 190021. Klinische opname \| \| 33290. Behandeling met de cardioverter. \| \| 39680. Perifeer infuus inbrengen. \| \| 190288. Verpleging. minder complexe zorg noodzakelijk in verband met medisch specialistische zorg in de thuissituatie. \| \| 33292. Resuscitatie. al of niet met defibrillatie. \| \| 39692. Preassessment opname. \| \| 33289. Resuscitatie. met intubatie. al of niet met defibrillatie (zie 033288 voor zonder intubatie). \| \| 39445. Injecties. met uitzondering van injecties ten behoeve van locale anesthesie. \| \| 39851. Behandeling middels CPAP of BiPAP. \| \| 33603. Venasectie. \| \| 33288. Resuscitatie. zonder intubatie. al of niet met defibrillatie (excl. met intubatie zie 033289). \| \| 39604. Inbrengen arteriële lijn. \| \| 39603. Inbrengen centrale veneuze lijn. \| \| 231902. Verpleegdag kaakchirurgie. \| \| 39682. Inbrengen voedingskatheter of centrale veneuze katheter niet gerelateerd aan een operatie. \| |
|  | Imaging general | \| 85090. MRI thorax(wand). mamma en mediastinum. \| \| --- \| \| 87042. CT onderzoek van het abdomen. retroperitoneum. inclusief inbegrepen orale en/of rectale contraststof. met of onder toediening van een intraveneus contrastmiddel. \| \| 87090. MRI abdomen. \| \| 87070. Echografie van de buikorganen. \| \| 81342. CT onderzoek van de hersenen en/of schedel met of zonder intraveneus contrastmiddel. \| \| 89070. Echografie onderste extremiteit(en). \| \| 39210. Mediastinoscopie. \| \| 86042. CT onderzoek van de luchtwegen. met of zonder intraveneus contrastmiddel. \| \| 120500. PET partieel (neurologisch. cardiologisch). \| \| 120501. PET WB (whole body). oncologie. \| \| 84070. Echografie van de bovenste extremiteit(en). \| \| 87097. MRI abdomen (excl. rectum. zie 087096). \| \| 80033. Inbrengen centrale lijn onder echo- of röntgengeleide. \| \| 80033. Inbrengen centrale lijn onder echo- of röntgengeleide. \| \| 80077. Diagnostische punctie of biopsie van niet palpabele afwijkingen of organen. onder echografische controle. \| \| 83042. CT onderzoek van de wervelkolom. \| \| 81093. MRI hersenen - standaard. \| \| 86802. Radiologisch onderzoek ribben en/of sternum. \| \| 87002. Radiologisch buikoverzichtsonderzoek. liggend en/of staand. een of meerdere richtingen. \| \| 85093. MRI thorax(wand) en mediastinum (excl. mamma. zie 085091). \| \| 39492. Echografie van de buikorganen. \| \| 39492. Echografie van de buikorganen. \| \| 84202. Radiologisch onderzoek scapula en/of clavicula en/of schoudergewricht en/of bovenarm. \| \| 39510. Aortografie. \| \| 82970. Echografie van de schildklier en/of hals. \| \| 83390. MRI lumbosacrale wervelkolom. \| \| 86902. Mammografie. al of niet met contrast in melkgangen (excl. mammografie - 3D. zie 086941). \| \| 82002. Radiologisch onderzoek aangezichtsschedel of deel ervan - neusbijholten inclusief sphenoid respectievelijk adenoid. \| \| 89602. Radiologisch onderzoek enkel en/of voet(wortel) en/of tenen. \| \| 81092. MRI hersenen - met contrast. \| \| 83290. MRI thoracale wervelkolom. \| \| 83202. Radiologisch onderzoek thoracale wervelkolom of deel ervan. \| \| 89879. Beoordeling radiologisch onderzoek voor derden. \| \| 89402. Radiologisch onderzoek knie en/of onderbeen. \| \| 82042. CT onderzoek van de aangezichtsschedel. met of zonder intraveneus contrast. \| \| 39498. Echografie met injectie. \| \| 83302. Radiologisch onderzoek lumbosacrale wervelkolom inclusief overzichtsopname sacroiliacale gewrichten. \| \| 84602. Radiologisch onderzoek pols en/of hand en/of vingers. \| \| 83190. MRI cervicale wervelkolom en/of hals inclusief craniovertebrale overgang. \| \| 34388. Endo-echografie ter beoordeling bovenbuikorganen. inclusief eventuele biopten. \| \| 89202. Radiologisch onderzoek bekken. respectievelijk heupgewricht. \| \| 86970. Echografie van mamma. \| \| 88920. Radiologisch onderzoek abdominale aorta inclusief bij dit onderzoek afgebeelde zijtakken en beenarteriën. \| \| 80057. Diagnostische punctie of biopsie van niet palpabele afwijkingen of organen. onder röntgencontrole. \| \| 89142. CT onderzoek van de onderste extremiteiten. met of zonder intraveneus contrast. \| \| 82505. Volledige gebitsstatus of panoramixopname. \| \| 87111. Radiologisch onderzoek slokdarm. \| \| 89090. MRI heup(en)/ onderste extremiteit(en). \| \| 81670. Echografie carotide. al dan niet inclusief haematotachografisch onderzoek van de cerebropetale vaten. \| \| 88620. Radiologisch onderzoek arteria uterina. \| \| 34386. Echo-oesofagoscopie. inclusief eventuele biopten. \| \| 81002. Radiologisch onderzoek hersenschedel of deel ervan inclusief neusbeen. \| \| 239455. Röntgengebitsonderzoek. ongeacht aantal en soort opnamen en inclusief eventuele controlefoto('s) - maximaal éénmaal per dag te declareren. \| \| 80032. Lokale injectie medicatie onder echo- of röntgengeleide. \| \| 89042. CT van het bekken inclusief inbrengen orale en/of rectale contraststof. Met of zonder toediening van een intraveneus contrastmiddel. \| \| 39899. Actigrafie. \| \| 84042. CT onderzoek van de bovenste extremiteit(en). met of zonder intraveneus contrast. \| \| 81089. MRI hersenen bij epilepsie - uitgebreid. \| \| 87048. Abcesdrainage met CT. \| \| 84402. Radiologisch onderzoek elleboog en/of onderarm. \| \| 83102. Radiologisch onderzoek cervicale wervelkolom of deel ervan. \| \| 89302. Radiologisch onderzoek bovenbeen. \| \| 80047. Diagnostische punctie of biopsie van niet palpabele afwijkingen of organen. onder CT-controle. \| \| 88120. Radiologisch onderzoek arteria renalis. ongeacht aantal arteriën per nier. \| \| 87078. Abcesdrainage met echografie. \| \| 87258. Inbrengen maag- of duodenumsonde. \| \| 82490. MRI achterste schedelgroeve. \| \| 80080. Volledig botdensitometrisch onderzoek met DEXA-apparatuur. ongeacht het aantal onderzochte anatomische gebieden en ongeacht het aantal zittingen. \| \| 83615. Inbrengen van intrathecaal of intra-articulair contrast door de radioloog. t.b.v. MRI of CT. \| \| 39918. Fundusfotografie in het kader van aandoeningen van het netvlies. exclusief screening op diabetische retinopathie (zie 039917). \| \| 82202. Skeletvrije opname oogbol en/of localisatie corpus alienum in oog(kas). \| \| 239452. Kostendeel röntgengebitsonderzoek en/of röntgenschedelonderzoek. elk maximaal éénmaal per dag te declareren. geleverd door het ziekenhuis of kaakchirurgen. \| \| 80001. Alleen doorlichten tijdens repositie fracturen of localisatie corpus alienum (waaronder röntgencontrole bij inbrengen pen in enkel. elleboog e.d.. doorlichting op OK. bewusteloze ongevalspatiënten). \| \| 39485. Echografie à-vue in verband met zwangerschap. \| \| 39485. Echografie à-vue in verband met zwangerschap. \| \| 83002. Radiologisch onderzoek gehele wervelkolom inclusief sacrum en os coccigis en overzichtsopname sacroiliacale gewrichten. \| \| 86202. Radiologisch onderzoek larynx en trachea inclusief struma-onderzoek al of niet met oesofaguscontrast. \| \| 83402. Speciaal gericht radiologisch onderzoek sacroiliacale gewrichten en/of os coccygis. \| \| 239462. Maken röntgenfoto (2D) ten behoeve van gebitsonderzoek en/of schedelonderzoek. elk maximaal éénmaal per dag te declareren. \| \| 39879. Echografie zenuwen en spieren. \| \| 87091. MRI lever. \| |
|  | Invasive/surgery general | \| 39881. Urologische behandeling dilataties en catheterisaties. \| \| --- \| \| 32660. Grote borstwandresectie in verband met een doorgegroeide maligniteit. \| \| 39693. Klinisch peri-operatieve zorg. \| \| 39696. Preassessment. \| \| 39210. Mediastinoscopie. \| \| 38942. Klinische wondexcisie en wondtoilet. Onder wondexcisie en wondtoilet wordt verstaan locaalanesthesie. inspectie. reiniging. excisie en/of hechting van de wond(en). \| \| 34620. Diagnostische endoscopie van oesofagus. maag en/of duodenum met behulp van flexibele endoscoop. eventueel inclusief biopten of antroduodenale manometrie. \| \| 32600. Behandeling met zuigdrainages van de pleuraholte en het mediastinum. \| \| 38952. Verwisselen wondbedekker t.b.v. vacuüm therapie. \| \| 39680. Perifeer infuus inbrengen. \| \| 39132. Procedurele sedatie en analgesie (PSA) buiten de operatiekamer. \| \| 33672. Percutane angioplastiek niet-coronaire perifere arteriën (zie 033351 voor niet-coronaire centrale arteriën). \| \| 32484. Therapeutische bronchoscopie. zoals verwijderen van corpora alinea. afzuigen van secretie of installatie van medicamenten. \| \| 30532. Peri-arteriële sympathectomie. \| \| 39090. Algehele anesthesie bij specialistisch onderzoek en bij verrichtingen. waarvoor geen anesthesie staat vermeld. \| \| 34394. Therapeutische endoscopie van oesofagus. maag of duodenum. \| \| 38824. Verwijdering plaat en schroeven uit een bot. \| \| 32684. Diagnostische pleurapunctie. \| \| 234041. Operatieve verwijdering van één of meerdere gebitselementen of één of meerdere radices of een corpus aliënum per kaakhelft - met splijten van het mucoperiost. \| \| 32603. Het spoelen van een empyeemholte door middel van een intra-thoracale zuigdrain of via een reeds voorhanden zijnde drain. met of zonder inbrengen van medicamenten. \| \| 38805. Excochleatie en/of sequestrotomie van middelgrote beenderen. \| \| 38805. Excochleatie en/of sequestrotomie van middelgrote beenderen. \| \| 84025. Flebografie van een arm inclusief de benodigde venapunctie. \| \| 33699. Percutane angioplastiek niet-coronaire perifere vaten (zie 033350 voor niet-coronaire centrale vaten en 033491 voor cerebropetale vaten). \| \| 39941. Tracheaspoeling en/of maagheveling. \| \| 38414. Resectie van de 1e rib bij costaclaviculair compressiesyndroom of van een van de overige ribben. \| \| 32203. Tracheotomie. \| \| 38806. Openbeitelen voor diagnostische doeleinden van grote beenderen. zoals femur. humerus. bekken en wervellichamen. \| \| 39445. Injecties. met uitzondering van injecties ten behoeve van locale anesthesie. \| \| 39034. Transpositie van fascie. \| \| 38953. Vacuüm therapie bij wondbehandeling. inclusief initieel aanbrengen wondbedekker. klinisch. per dag. \| \| 39606. Uitleiden algemene anesthesie. \| \| 39605. Inleiden algemene anesthesie. \| \| 39607. Anesthesie controle door anesthesist op verkoever. \| \| 39607. Anesthesie controle door anesthesist op verkoever. \| \| 33490. Excisiebiopsie van de arteria temporalis. \| \| 38945. Wondbehandeling wond met wondrandexcisie (o.a. necrotomie. debridement) of wond > 5 cm lengte zonder wondrandexcisie. met onderzoek en schoonmaken en hechten of lijmen. incl. evt. plaats. verdoven. \| \| 38823. Verwijdering krammen na een epiphysiodese. \| \| 35502. Therapeutische ascitespunctie. \| \| 39860. Chromocystoscopie. \| \| 39859. Cysto-urethroscopie. \| \| 38428. Refixatie sternum. \| \| 32281. Diagnostische directe laryngoscopie. inclusief eventuele proefexcisie(s). \| \| 35355. Cholecystectomie per laparoscoop. inclusief eventueel peroperatief te verrichten cholangiogram. \| \| 33224. Neuroprotectieve hypothermiebehandeling. \| \| 33350. Percutane transluminale angioplastiek niet-coronaire centrale vaten (zie 033699 voor niet-coronaire perifere vaten en 033491 voor cerebropetale vaten). \| \| 32540. Pleuro-pneumonectomie. open procedure. \| \| 30555. Percutane thermolaesie van het dorsale ganglion. ongeacht het aantal. inclusief prognostische blokkade. lumbosacraal. \| \| 238044. Operatieve behandeling van een meervoudige mandibula-fractuur of van een maxilla- of zygoma-fractuur. \| \| 39887. Intravesicale instillatie. \| \| 38407. Percutane botboring. zoals uit het os ileum of de spina van de wervels. uitgezonderd de sternumpunctie. \| \| 34452. Laparoscopische gastric bypass operatie (oa. biliopancreatische deviatie. duodenale switch). \| \| 30540. Neurolytische blokkade van een of meer perifere zenuwen. \| \| 30552. Percutane facetdenervatie met behulp van thermolaesies onder beeldvormende techniek. inclusief prognostische blokkade. lumbosacraal. \| \| 38590. Amputatie bovenbeen. \| \| 38641. Uitgebreide artrotomie. patellectomie. cheilectomie. synovectomie en kruisbandplastiek van de knie. \| |
|  | Other | \| 190218. Verpleegdag. \| \| --- \| \| 190204. Verpleegdag. \| \| 198209. Groepsconsult maatschappelijk werk. \| \| 198207. Behandelcontact maatschappelijk werk. \| \| 198206. Herhaalconsult maatschappelijk werk. \| \| 198205. Intake maatschappelijk werk. \| \| 198208. Teleconsult maatschappelijk werk. \| \| 39132. Procedurele sedatie en analgesie (PSA) buiten de operatiekamer. \| \| 120400. Behandeling hyperthyreoidie met I-131. \| \| 190133. Micu transport >= 2 uur. \| \| 39939. Aerosolbehandeling. \| \| 39830. Het inbrengen van een verblijfnaald ter verkrijging van arterieel bloed voor onderzoek naar pO2. pH en pCO2 voor en tijdens inademen van zuurstof. \| \| 39678. Transport patiënt buiten het OK-complex. \| \| 190038. Verblijf vervallen ziekenhuisindicatie. niet verpleeghuis. \| \| 39577. Comprehensive Geriatric Assessment (CGA). \| \| 38953. Vacuüm therapie bij wondbehandeling. inclusief initieel aanbrengen wondbedekker. klinisch. per dag. \| \| 39831. Het inbrengen van een verblijfnaald ter verkrijging van arterieel bloed voor onderzoek naar pO2 en pCO2 in rust en tijdens inspanning. zonodig ook na inademen van zuurstof. \| \| 39992. Lichttherapie. al of niet ondersteund door medicamenteuze fotosensibiliserende therapie. per behandeling. \| \| 194171. Consultaties - overleg (inter)collegiaal - medisch psycholoog. \| \| 190307. Osteosynthesemateriaal wervelkolom. \| \| 39694. Voorbereiding wegens overplaatsing naar tertiair centrum (stabiliseren patiënt voor transport. formuleren overdracht aan IC-team). \| \| 38940. Eenmalig traumatologisch consult zonder verwijzing en specialistische nabehandeling. \| \| 39233. Screening longtransplantatie ontvanger. \| |
|  | Administrative | \| 79991. Ordertarief klinisch-chemische en microbiologische laboratoriumonderzoeken. inclusief bloedafname. \| \| --- \| \| 79990. Toeslag op ordertarief bij decentrale afname van patiëntmateriaal. \| \| 194161. Nieuwe inschrijving (verwijzing. registratie. informatie. dossier) - medisch psycholoog. \| \| 190034. Afwezigheidsdag \| \| 79988. Registratietarief (inclusief oproep) in het kader van multidisciplinaire zorgverlening bij chronische aandoeningen. \| |
| Outpatient Care | Consult | \| 190011. Eerste polikliniekbezoek. \| \| --- \| \| 190012. Polikliniekbezoek. niet zijnde een eerste polikliniekbezoek. dat leidt tot opening van een nieuwe DBC. \| \| 190025. Teleconsult (exclusief screen to screen beeldcontact. zie 190019). \| \| 190014. Doelgerichte telefonische consultatie van een poortspecialist door een patiënt bij een al geopende DBC ter vervanging van een fysiek consult. \| \| 198203. Teleconsult verpleegkundig specialist. \| \| 198203. Teleconsult verpleegkundig specialist. \| \| 79992. Huisbezoektarief klinisch-chemische en microbiologische laboratoriumonderzoeken. \| \| 79992. Huisbezoektarief klinisch-chemische en microbiologische laboratoriumonderzoeken. \| \| 290164. Reguliere behandeling extramurale diëtetiek (per kwartier). \| \| 234003. Consult. \| \| 70028. Consult bij patiënt op verzoek van een ondersteunend specialist door een poortspecialist bij een al geopende DBC in verband met direct patiënt gerelateerde vraagstelling. \| \| 193026. Telefonische zitting (fysiotherapie). \| \| 70027. Doelgerichte consultatie van ondersteunend specialist door poortspecialist bij al geopende DBC ivm direct patiënt gerelateerde vraagstelling. telefonisch of face-to-face. zonder aanwezigheid patiënt. \| |
|  | Rehabilitaton | \| 193140. Behandelsessie FIT - in het kader van FIT module hartrevalidatie. \| \| --- \| \| 193001. Individuele zitting reguliere fysiotherapie. \| \| 193126. Intakecontact. \| \| 193127. Informatiemodule. \| \| 193129. FIT-module > tien sessies. \| \| 193029. Intake en onderzoek na verwijzing (fysiotherapie) \| \| 193025. Lange zitting voor een aantal specifieke aandoeningen (fysiotherapie). \| \| 39898. Coördinatie bij hart- of longrevalidatie. \| \| 193128. FIT-module < tien sessies. \| \| 193021. Individuele zitting reguliere logopedie. \| \| 190961. Fysiotherapie - direct patiëntgebonden handelen - revalidatie. \| \| 193012. Individuele zitting (ergotherapie). \| \| 190960. Arts. verpleegkundig specialist of physician assistant - direct patiëntgebonden handelen - revalidatie. \| \| 193141. Behandelsessie PEP - in het kader van PEP module hartrevalidatie. \| \| 192971. Individuele zitting afasie (logopedie). \| \| 190964. Maatschappelijk werk - direct patiëntgebonden handelen - revalidatie. \| \| 190940. Fysiotherapie - indirect patiëntgebonden handelen - revalidatie. \| \| 190940. Fysiotherapie - indirect patiëntgebonden handelen - revalidatie. \| \| 190943. Maatschappelijk werk - indirect patiëntgebonden handelen - revalidatie. \| \| 190943. Maatschappelijk werk - indirect patiëntgebonden handelen - revalidatie. \| \| 193130. PEP-module. \| \| 190977. Geestelijke verzorging - direct patiëntgebonden handelen - revalidatie. \| \| 190944. Psychologie - indirect patiëntgebonden handelen - revalidatie. \| \| 193088. Toeslag voor buiten reguliere werktijden (fysiotherapie). \| \| 190962. Ergotherapie - direct patiëntgebonden handelen - revalidatie. \| |
|  | Visit | \| 190013. Herhaal-polikliniekbezoek. \| \| --- \| \| 190060. Eerste polikliniekbezoek. \| \| 190011. Eerste polikliniekbezoek. \| \| 33285. Poliklinische controle op de werking van de pacemaker. inclusief het eventueel gebruik van de cardioverter. \| \| 190288. Verpleging. minder complexe zorg noodzakelijk in verband met medisch specialistische zorg in de thuissituatie. \| \| 38941. Poliklinische wondexcisie en wondtoilet zonder verwijzing. Onder wondexcisie en wondtoilet wordt verstaan locaalanesthesie. inspectie. reiniging. excisie en/of hechting van de wond(en). \| \| 190289. Verpleging. complexe zorg noodzakelijk in verband met medisch specialistische zorg in de thuissituatie. \| \| 38943. Poliklinische wondexcisie en wondtoilet na verwijzing. Onder wondexcisie en wondtoilet wordt verstaan locaalanesthesie. inspectie. reiniging. excisie en/of hechting van de wond(en). \| |
|  | Other | \| 38954. Vacuüm therapie bij wondbehandeling. inclusief initieel aanbrengen wondbedekker. poliklinisch. per dag. \| \| --- \| \| 38954. Vacuüm therapie bij wondbehandeling. inclusief initieel aanbrengen wondbedekker. poliklinisch. per dag. \| \| 38941. Poliklinische wondexcisie en wondtoilet zonder verwijzing. Onder wondexcisie en wondtoilet wordt verstaan locaalanesthesie. inspectie. reiniging. excisie en/of hechting van de wond(en). \| \| 39133. Telemonitoring. \| |
| Other | Material | \| 32671. Perfusie per OK sessie. uitgevoerd of standby (inclusief materiaal. inzet apparatuur en perfusiepersoneel). \| \| --- \| \| 39102. Circulair gips voor hand en onderarm. \| \| 38894. Verwijderen klein gips. \| |
|  | Dialysis | \| 190156. IC dialysetoeslag. \| \| --- \| \| 190144. IC dialysetoeslag groep 3. Registreren naast IC behandeldag (190141). indien op een IC behandeldag op enig moment sprake is van nierdialyse onder eindverantwoordelijkheid van een medisch specialist. \| \| 192051. Hemodialyse. \| \| 39977. De eerste acute nierfunctievervangende therapie. bij acuut nierlijden. intoxicaties etc. \| |
|  | Medication | \| 191957. Novoseven. per toedieningeenheid van 2 mg bij de indicaties welke niet zijn opgenomen in de NZa Prestatie- en tarieventabel stollingsfactoren. \| \| --- \| \| 191977. Cofact. per toedieningeenheid van 500 E bij de indicaties welke niet zijn opgenomen in de NZa Prestatie- en tarieventabel stollingsfactoren. \| \| 193349. Etanercept. toedieningsvorm injectiepoeder. per gebruikte eenheid van 1 mg bij indicaties welke bij deze stofnaam zijn opgenomen in de NZa Prestatie- en tarieventabel add-on geneesmiddelen. \| \| 193309. Infliximab. toedieningsvorm infusiepoeder. per gebruikte eenheid van 1 mg bij indicaties welke bij deze stofnaam zijn opgenomen in de NZa Prestatie- en tarieventabel add-on geneesmiddelen. \| \| 191930. Haemocomplettan P. per toedieningeenheid van 1 g bij de indicaties welke niet zijn opgenomen in de NZa Prestatie- en tarieventabel stollingsfactoren. \| \| 193347. Adalimumab. toedieningsvorm injectievloeistof. per gebruikte eenheid van 1 mg bij indicaties welke bij deze stofnaam zijn opgenomen in de NZa Prestatie- en tarieventabel add-on geneesmiddelen. \| \| 39145. Verstrekking chemo-immunotherapie per infuus of per injectie. \| \| 193308. Paclitaxel. toedieningsvorm infusievloeistof. per gebruikte eenheid van 1 mg bij indicaties welke bij deze stofnaam zijn opgenomen in de NZa Prestatie- en tarieventabel add-on geneesmiddelen. \| \| 193440. Abatacept. toedieningsvorm injectievloeistof. per gebruikte eenheid van 1 mg bij indicaties welke bij deze stofnaam zijn opgenomen in de NZa Prestatie- en tarieventabel add-on geneesmiddelen. \| \| 194537. Melfalan. per gebruikte eenheid van 1 mg bij indicaties welke niet bij deze stofnaam zijn opgenomen in de NZa Prestatie- en tarieventabel add-on geneesmiddelen. \| \| 39163. Intraveneuze verstrekking van bisfosfonaten. \| \| 193338. Voriconazol. toedieningsvorm tablet. per gebruikte eenheid van 1 mg bij indicaties welke bij deze stofnaam zijn opgenomen in de NZa Prestatie- en tarieventabel add-on geneesmiddelen. \| \| 191976. Cofact. per toedieningeenheid van 250 E bij de indicaties welke niet zijn opgenomen in de NZa Prestatie- en tarieventabel stollingsfactoren. \| \| 193541. Anidulafungine. per gebruikte eenheid van 1 mg bij indicaties welke niet bij deze stofnaam zijn opgenomen in de NZa Prestatie- en tarieventabel add-on geneesmiddelen. \| |
|  | Administrative | \| 79990. Toeslag op ordertarief bij decentrale afname van patiëntmateriaal. \| \| --- \| \| 119052. Schriftelijke informatieverstrekking aan bedrijfsarts. verzekeringsarts of (via de patiënt aan) het centraal bureau rijvaardigheidsbewijzen (CBR). \| |
