# Supplementary material for: Utilization of diagnostic resources and costs in patients with suspected cardiac chest pain
Source: Eur Heart J Qual Care Clin Outcomes. 2020 Aug 18;7(6):583–90. doi: 10.1093/ehjqcco/qcaa064 (PMC9172873; doi:10.1093/ehjqcco/qcaa064)
Supplement: qcaa064_Supplementary_Data [file qcaa064_supplementary_data.zip › Table 1 supplemental.docx]

**SUPPLEMENTAL MATERIAL**

**Supplemental Table 1,** Overview of diagnostic codes which are categorized as ‘Ischemic Cardiac History’

| **Ischemic Cardiac History** | **Diagnostic codes** |
| --- | --- |
| Unstable Angina Pectoris | 0320.11.203 / ICD- 10 code I20.0 |
| ST-elevation Myocardial Infarction (STEMI) | 0320.11.204 / ICD- 10 code I21.9 |
| Non ST elevation myocardial infarction (NSTEMI) | 0320.11.205 / ICD- 10 code I21.4 |
| Follow up after acute coronary syndrome | 0320.11.801 / ICD- 10 code Z86.7 |
| Follow up after transluminal coronary angioplasty (PTCA) | 0320.11.802 / ICD- 10 code Z09.0 |
| Coronary artery bypass grafting (CABG) or ablation | 0320.11.802 / ICD- 10 code Z09.0 |
| Follow up after heart operation | 0320.11.810 / ICD- 10 code Z09.0 |

| **Cardiac Diseases** | **Diagnostic codes** |
| --- | --- |
| Acute Heart Failure | 0320.11.301 / ICD- 10 code I51.9 |
| Chronic Heart Failure | 0320.11.302 / ICD- 10 code I50.9 |
| Atrial Fibrillation | 0320.11.401 / ICD- 10 code I48.9 |
| Other Supraventricular Rhythm Disorders | 0320.11.402 / ICD- 10 code I47.1 |
| Ventricular Rhythm Disorder | 0320.11.403 / ICD- 10 code I47.2 |
| Impulse And Conduction Disorder | 0320.11.404 / ICD- 10 code I45.5 of I45.9 |
| Other Heart Rhythm disorders | 0320.11.409 / ICD- 10 code I49.8 |
| MAZE procedure | 0320.11.2525 / ICD- 10-PCS according to procedure |
| Arterial malformation or stenosis | 0320.11.601 / ICD- 10 code I71.0 or I71.9 |
| Venous malformation | 0320.11.602 / ICD- 10 code I82.9 |
| Other vascular disorders | 0320.11.609 / ICD- 10 code I51.6 |
| Pericarditis | 0320.11.701 / ICD- 10 code I31.9 |
| Endocarditis | 0320.11.702 / ICD- 10 code I38 |
| Other cardiac infections | 0320.11.709 / ICD- 10 code A49.8 |

**Supplemental Table 2.** Overview of diagnostic codes which are categorized as ‘Cardiac Diseases’
